# Temperature-Dependent Nitrous Oxide/Carbon Dioxide Preferential Adsorption in a Thiazolium-Functionalized NU-1000 Metal–Organic Framework

**DOI:** 10.1021/acsami.1c21437

**Published:** 2021-12-02

**Authors:** Giorgio Mercuri, Marco Moroni, Simona Galli, Giulia Tuci, Giuliano Giambastiani, Tongan Yan, Dahuan Liu, Andrea Rossin

**Affiliations:** † Istituto di Chimica dei Composti Organometallici (ICCOM-CNR), Via Madonna del Piano 10, 50019 Sesto Fiorentino, Italy; ‡ Dipartimento di Scienza e Alta Tecnologia, Università dell’Insubria, Via Valleggio 11, 22100 Como, Italy; § Institute of Chemistry and Processes for Energy, Environment and Health (ICPEES), UMR 7515 CNRS-University of Strasbourg (UdS), 25, rue Becquerel, 67087 Strasbourg Cedex 02, France; ∥ State Key Laboratory of Organic-Inorganic Composites, Beijing University of Chemical Technology, Beijing 100029, China

**Keywords:** metal−organic frameworks (MOFs), porous materials, zirconium(IV), thiazolium salts, carbon dioxide adsorption, nitrous oxide adsorption, powder X-ray diffraction (PXRD), grand canonical Monte Carlo (GCMC) simulations, molecular dynamics (MD) simulations

## Abstract

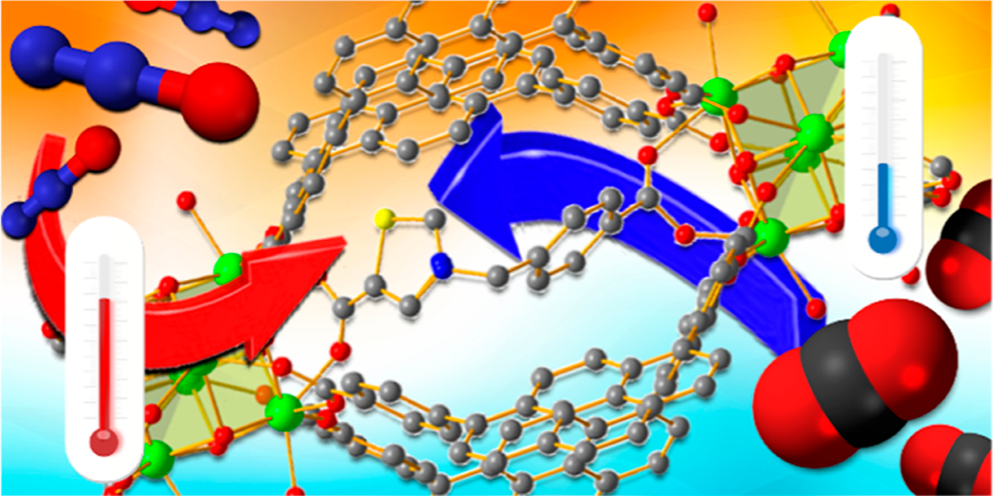

Solvent-assisted ligand incorporation (SALI) of the ditopic linker 5-carboxy-3-(4-carboxybenzyl)thiazolium bromide [(**H**_**2**_**PhTz**)Br] into the zirconium metal–organic framework **NU-1000** [Zr_6_O_4_(OH)_8_(H_2_O)_4_(TBAPy)_2_, where NU = Northwestern University and H_4_TBAPy = 1,3,6,8-tetrakis(*p*-benzoic-acid)pyrene], led to the SALIed **NU-1000-PhTz** material of minimal formula [Zr_6_O_4_(OH)_6_(H_2_O)_2_(TBAPy)_2_(**PhTz**)]Br. **NU-1000-PhTz** has been thoroughly characterized in the solid state. As confirmed by powder X-ray diffraction, this material keeps the same three-dimensional architecture of **NU-1000** and the dicarboxylic extra linker bridges adjacent [Zr_6_] nodes *ca.* 8 Å far apart along the crystallographic *c*-axis. The functionalized MOF has a BET specific surface area of 1560 m^2^/g, and it is featured by a slightly higher thermal stability than its parent material (*T*_dec_ = 820 vs. 800 K, respectively). **NU-1000-PhTz** has been exploited for the capture and separation of two pollutant gases: carbon dioxide (CO_2_) and nitrous oxide (N_2_O). The high thermodynamic affinity for both gases [isosteric heat of adsorption (*Q*_st_) = 25 and 27 kJ mol^–1^ for CO_2_ and N_2_O, respectively] reasonably stems from the strong interactions between these (polar) “stick-like” molecules and the ionic framework. Intriguingly, **NU-1000-PhTz** shows an unprecedented temperature-dependent adsorption capacity, loading more N_2_O in the 298 K ≤ *T* ≤ 313 K range but more CO_2_ at temperatures falling out of this range. Grand canonical Monte Carlo simulations of the adsorption isotherms confirmed that the preferential adsorption sites of both gases are the triangular channels (micropores) in close proximity to the polar pillar. While CO_2_ interacts with the thiazolium ring in an “end-on” fashion through its O atoms, N_2_O adopts a “side-on” configuration through its three atoms simultaneously. These findings open new horizons in the discovery of functional materials that may discriminate between polluting gases through selective adsorption at different temperatures.

## Introduction

**Figure 1:**
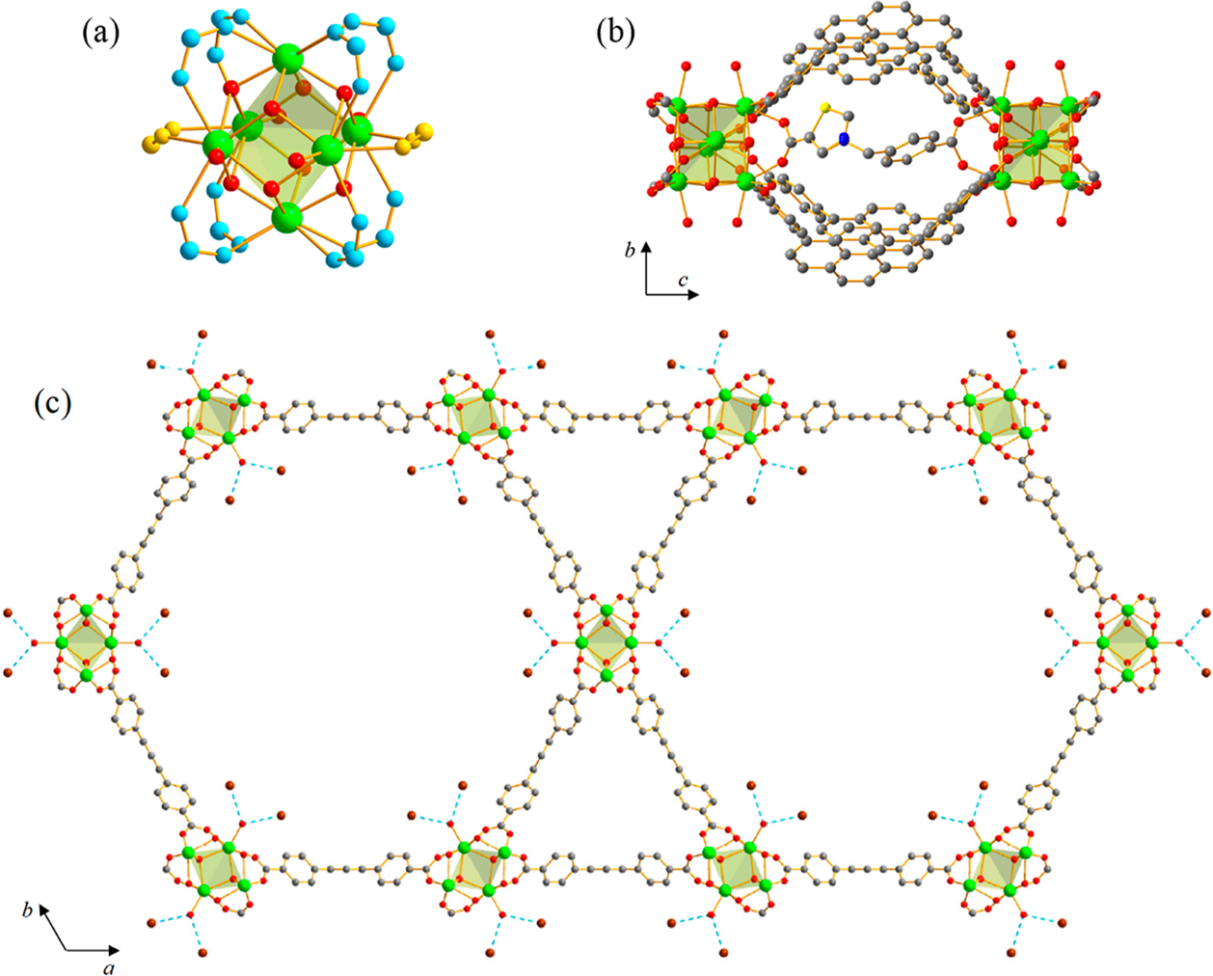
Representation of the crystal structure of **NU-1000-PhTz**: (a) Zr-based oxo–hydroxo cluster coordinated by the carboxylate groups belonging to the TBAPy^4–^ ligands (blue atoms) and to **PhTz**^**–**^ (yellow atoms); (b) bridging coordination mode of **PhTz**^**–**^ in the ∼8 Å cavity along the *c-*axis; (c) crystal packing viewed along the [001] crystallographic direction. Hydrogen bonds involving OH^–^/H_2_O and Br^–^ are highlighted with blue dashed lines. Oxygen atoms representing the smeared electron density and hydrogen atoms are omitted for the sake of clarity. Atom color code: carbon, gray; bromine, brown; nitrogen, blue; oxygen, red; sulfur, yellow; zirconium, light green.

**Scheme 1:**
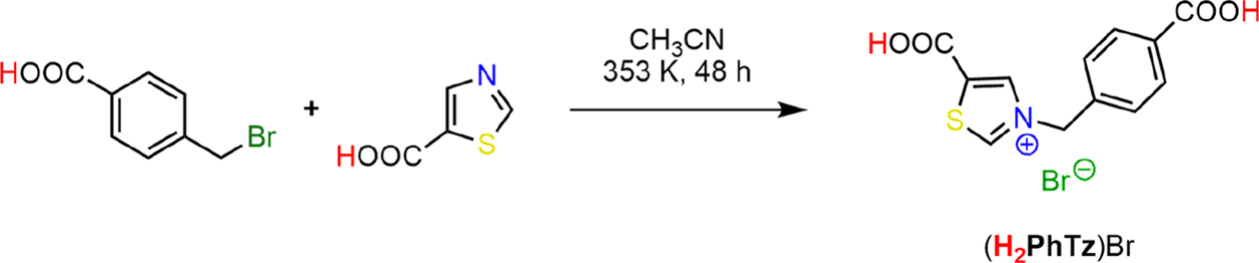
Synthesis of (**H**_**2**_**PhTz**)Br

The synthetic tools available for the preparation of metal–organic frameworks (MOFs) have progressively increased in number in more recent years. MOFs are crystalline materials composed of inorganic nodes coordinated *via* multitopic organic linkers, with a wide structural variety coming from the virtually infinite “Tinkertoy” combinations of their constituting building units.^1−4^ Initially, the solvothermal/hydrothermal approach (i.e., mixing metal salts and polytopic linkers in a high-boiling polar solvent and treating the mixture at high temperature under autogenous pressure in sealed autoclaves) was the most popular synthetic methodology to prepare new MOFs. After the serendipitous discovery of the ability of zirconium MOFs to participate in linker exchange or inclusion reactions while keeping their crystal structure intact, new perspectives on MOF synthesis have come up ahead. Indeed, this has led to the so-called solvent-assisted ligand exchange^5−7^ and solvent-assisted ligand incorporation (SALI) postsynthetic methodologies.^8−11^ The former is now highly exploited to prepare mixed-ligand MOFs through partial exchange of the pre-existing linker with new ones dissolved in a solution in contact with a suspended MOF powder at a high temperature. The latter approach stems from the existence, in some [Zr_6_] octahedral nodes, of monodentate hydroxo/aquo ligands that are prone to react with the COOH groups of the incoming carboxylate-based linkers that eventually replace them on the metallic node through a simple condensation reaction (and concomitant water elimination). Thus, SALI is a powerful synthetic tool to insert new species in pre-existing MOFs with the aim of creating new materials with enhanced properties. One of the most iconic zirconium MOFs is **NU-1000** (NU = Northwestern University)^12^ with its [Zr_6_(μ_3_-OH)_4_(μ_3_-O)_4_(OH)_4_(H_2_O)_4_]^8+^ nodes and tetratopic pyrene-based linkers [H_4_TBAPy = 1,3,6,8-tetrakis(*p*-benzoic acid)pyrene]. **NU-1000** is particularly suitable for SALI because the hydroxo/aquo ligands dangling from the eight-connected [Zr_6_] nodes are oriented toward both the 30 Å wide hexagonal channels (along the crystallographic *a*-axis and *b*-axis) and the smaller 8 Å cavities (along the crystallographic *c*-axis). Consequently, after −OH/–OH_2_ ligand replacement, up to four additional carboxylate groups may be added to the metallic nodes to complete the Zr^IV^ coordination sphere and form a 12-connected [Zr_6_] cluster, with a concomitant topology change. The resulting **NU-1000-FG** material (FG = functional group) is featured by new chemicophysical properties that depend on those of the extra linker added and on the SALI extent. Previous works have already shown the great potentiality of the technique in this context.^11,13−15^ Following the research line of our group on the synthesis of MOF materials containing polar heterocyclic linkers for enhanced polluting gas capture and separation,^16−18^ we exploited SALI to prepare a new **NU-1000-FG** derivative suitable for both carbon dioxide (CO_2_) and nitrous oxide (N_2_O) adsorption. While some of these compounds have shown excellent performances in carbon dioxide storage^8,19^ with high absolute uptake under ambient temperature and pressure conditions and enhanced thermodynamic affinity compared to the parent **NU-1000**,^11,15,20^ to the best of our knowledge, no examples of **NU-1000-FG** MOFs exploited for nitrous oxide storage are known to date. N_2_O occurs in ever-increasing amounts in the atmosphere due to the industrial anthropogenic activity (nitric acid and adipic acid production), and it has been found to be a major scavenger of stratospheric ozone with the same degradative effect as that of chlorofluorocarbons. Being the third most important long-lived greenhouse gas after methane (CH_4_) and CO_2_, nitrous oxide substantially contributes to global warming with an extent comparable to that of CO_2_, albeit being present in much smaller concentration in the Earth atmosphere. On a per-molecule basis, nitrous oxide has *ca.* 300 times the atmospheric heat-trapping ability of carbon dioxide. Thus, it is important to design chemical sponges that capture N_2_O efficiently. From a chemical viewpoint, the two molecules are isoelectronic, share the same “stick-like” linear shape, and possess the same molecular weight (44 amu). On the other hand, N_2_O is not thermodynamically stable versus the elements; moreover, while CO_2_ is quadrupolar, N_2_O shows a small dipole moment (0.166 D), the anisotropic distribution of its electronic density being further enhanced by the existence of two resonance forms with integer charges: {N≡N^+^–O^–^ ↔ ^–^N=N^+^=O}. More in general, they show similarities and differences at the chemicophysical^21^ and biological^22^ levels. The inclusion of polar linkers within **NU-1000** should be beneficial for N_2_O uptake, as observed for CO_2_. Following this idea and our previous experience on the design of thiazole-containing polytopic carboxylates for MOF synthesis,^23−28^ we have prepared the ditopic thiazolium carboxylate salt 5-carboxy-3-(4-carboxybenzyl)thiazolium bromide (**H**_**2**_**PhTz**)Br (Scheme 1). This flexible dicarboxylic acid has been anchored to the **NU-1000** nodes *via* SALI in a bridging fashion between adjacent [Zr_6_] clusters *ca.* 8 Å far apart. The resulting **NU-1000-PhTz** MOF (Figure 1) has been characterized in the solid state and exploited for CO_2_ and N_2_O capture, showing an unexpected temperature-dependent N_2_O/CO_2_ preferential adsorption.

## Experimental Section

### Materials and Methods

All the chemicals and reagents employed were purchased from commercial suppliers and used as received without further purification. **NU-1000** was prepared according to the published procedure.^29^ For organic syntheses, solvents were purified through standard distillation techniques. Deuterated solvents (Sigma-Aldrich) were stored over 4 Å molecular sieves and degassed by three freeze–pump–thaw cycles before use. NMR spectra were recorded on a Bruker Avance 400 MHz spectrometer. ^1^H and ^13^C{^1^H} NMR chemical shifts are reported in parts per million (ppm) downfield of tetramethylsilane (TMS) and were calibrated against the residual resonance of the protiated part of the deuterated solvent. FT-IR spectra (KBr pellets) were recorded on a PerkinElmer Spectrum BX Series FTIR spectrometer, in the 4000–400 cm^–1^ range, with a 2 cm^–1^ resolution. Thermogravimetric analyses (TGAs) were performed under N_2_ flow (100 mL min^–1^) at a heating rate of 10 K min^–1^ on an EXSTAR TG Analyzer (TG-DTG) Seiko 6200. Elemental analyses were carried out using a Thermo FlashEA 1112 Series CHNS-O elemental analyzer with an accepted tolerance of ±2% on carbon (C), hydrogen (H), nitrogen (N), and sulfur (S). ESI-MS spectra were recorded by direct sample introduction (10 μL/min) in a Finnigan LTQ mass spectrometer (Thermo, San Jose, CA). The instrument was equipped with a conventional ESI source. The working conditions were the following: positive polarity–spray voltage, 5 kV; capillary voltage, 35 V; capillary temperature, 548 K; tube lens, 110 V. The sheath gas pressure was set at 10 au and the auxiliary gas pressure was kept at 3 au. For the acquisitions, the Xcalibur 2.0 software (Thermo) was used. DMSO solutions of (**H**_**2**_**PhTz**)Br (1 mg/mL) were diluted to 10 ng/μL with a MeOH/H_2_O 1:1 v/v solution. Powder X-ray diffraction (PXRD) qualitative measurements were carried out in the 2–50° 2θ region with a Panalytical X’PERT PRO diffractometer equipped with a diffracted beam Ni filter, a PIXcel^©^ solid-state detector, and a sealed X-ray tube (Cu Kα, λ = 1.5418 Å). Slits were used on both the incident beam (Soller slits aperture: 0.25°; divergence slits aperture: 0.5°) and the diffracted beam (antiscatter slit aperture: 7.5 mm). X-ray fluorescence (XRF) qualitative elemental analysis was performed on a powdered batch (*ca.* 10 mg) of **NU-1000-PhTz** with a Panalytical MINIPAL 2 instrument equipped with a Cr X-ray source. X-ray photoelectron spectroscopy (XPS) analyses were conducted in an ultrahigh vacuum (UHV) spectrometer equipped with a VSW Class WA hemispherical electron analyzer and a monochromatic Al Kα X-ray source (1486.6 eV) as the incident radiation. Survey and high-resolution spectra were recorded in constant pass energy mode (90 and 44 eV, respectively). Binding energy (BE) values for all spectra were calibrated using the C 1s sp^2^ component at 284.8 eV. Signal fitting was performed with the CasaXPS software using mixed Gaussian–Lorentzian curves.

### Synthesis of 5-Carboxy-3-(4-carboxybenzyl)thiazolium Bromide [(H_2_PhTz)Br]


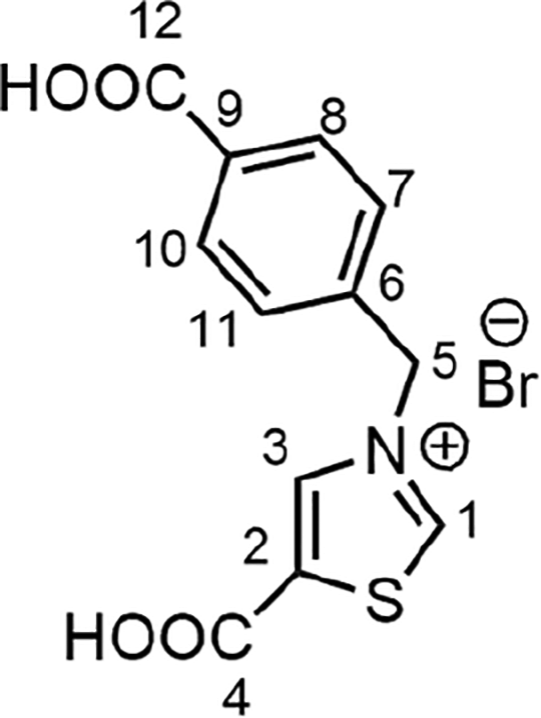
A stirred solution of thiazole-5-carboxylic acid (FW = 129.13 g/mol, 0.4 g, 3.1 mmol) and 4-bromomethyl benzoic acid (FW = 215.04 g/mol, 0.8 g, 3.7 mmol, 1.2 equiv) in acetonitrile (25 mL) was kept at 353 K for 48 h. During this time, an off-white solid formed and precipitated out of the solution. Afterward, the mixture was cooled down to ambient temperature, and acetonitrile was removed after decantation. The remaining solid was washed with acetone (3 × 10 mL) to remove any impurities or unreacted starting material. Finally, the solid was dried *in vacuo* to give pure (**H**_**2**_**PhTz**)Br as an off-white powder (yield: 0.9 g, 84% based on thiazole-5-carboxylic acid). ^1^H NMR (400 MHz, DMSO-*d*_6_, 298 K): δ (ppm) 10.54 (s, 1H, H^1^), 9.21 (s, 1H, H^3^), 7.99 (d, ^3^*J*_HH_ = 8.25 Hz, 2H, H^8,10^), 7.63 (d, ^3^*J*_HH_ = 8.25 Hz, 2H, H^7,11^), 5.87 (s, 2H, H^5^). ^13^C{^1^H} NMR (100 MHz, DMSO-*d*_6_, 298 K): δ (ppm) 167.49 (C^4^), 164.25 (C^1^), 160.16 (C^12^), 141.42 (C^3^), 138.86 (C^2^), 136.31 (C^9^), 132.05 (C^6^), 130.51 (C^8,10^), 129.49 (C^7,11^), 58.10 (C^5^). Elem. Anal. Calcd (%) for C_12_H_10_BrNO_4_S (FW = 344.18 g/mol): C, 41.88; H, 2.93; N, 4.07; S, 9.32. Found: C, 41.91; H, 2.96; N, 4.11; S, 9.30. IR (KBr pellet, cm^–1^): ν = 3067 [m, ν(C–H)_aromatic_], 2896 [m, ν(C–H)_aliphatic_], 1726 [s, ν(COO)], 1709 [s, ν(COO)], 1612, 1582 [m, ν(C=C)], 1420 (m), 1406 (s), 1377 (s), 1224 [s, δ(O–H)], 1151(m), 1108 (m), 796 [s, γ(C–H)]. ESI-MS: *m*/*z* = 264 (M-Br)^+^, 220 (M-Br–CO_2_)^+^, 176 (M-Br–2CO_2_)^+^. Single crystals of the zwitterionic neutral form **HPhTz** suitable for X-ray diffraction were obtained from a concentrated methanolic solution layered with acetonitrile at 298 K. The single-crystal X-ray diffraction data acquisition and treatment as well as the molecular structure are reported in the Supporting Information (Figures S1 and S2 and Table S1).

### Synthesis of [Zr_6_O_4_(OH)_6_(H_2_O)_2_(TBAPy)_2_(PhTz)]Br·8(H_2_O) (**NU-1000-PhTz**)

According to the general SALI procedure reported by Hupp, Farha et al.,^11,15^ the thiazolium bromide dicarboxylate salt (**H**_**2**_**PhTz**)Br (0.127 g, 0.370 mmol, 10 equiv) was added to a suspension of benzoate-free^30^**NU-1000** (0.080 g, 0.037 mmol) in a dry and degassed polar solvent mixture (total volume, 37 mL; acetonitrile/dimethylsulfoxide = 90:10 v/v). The reaction mixture was heated at 353 K for 24 h with occasional gentle swirling. After that time, the mixture was brought back to room temperature, and the precipitate was filtered over a 0.2 μm PTFE filter. The bright yellow solid residue of **NU-1000-PhTz** was sequentially washed with hot acetonitrile, acetone, and dichloromethane (3 × 20 mL each) and finally dried in air. Yield: 90 mg (94%, based on zirconium). The extent of thiazolium salt incorporation (one **PhTz**^**–**^ per [Zr_6_] node) was determined through both the structural characterization from PXRD (*vide infra*) and signal integration of the ^1^H NMR spectrum of the solution obtained after digesting the sample in a D_2_SO_4_/D_2_O/DMSO-*d*_6_ mixture and heating to 363 K for 2 h (see the Supporting Information and Figure S3). IR (KBr pellet, cm^–1^): ν = 1676 (sh), 1604 (m), 1542 (m) [ν(C=O)], 1419 (s), 1384 (s), 1275 (m), 1261 (s), 1182 (w), 1148 (w), 1106 (w), 784 (m), 716 (m), 659 (m).

### PXRD Structural Characterization

A powdered sample (∼50 mg) of **NU-1000-PhTz** was inserted in the cavity of a silicon-free background sample holder 0.2 mm deep (Assing Srl, Monterotondo, Italy) and analyzed by means of PXRD using a Bruker AXS D8 Advance vertical-scan θ/θ diffractometer, equipped with a sealed X-ray tube (Cu Kα, λ = 1.5418 Å), a Bruker Lynxeye linear position-sensitive detector, a Ni filter in the diffracted beam, and the following optical components: primary beam Soller slits (2.5°), fixed divergence slit (0.5°), and antiscatter slit (8 mm). The generator was operated at 40 kV and 40 mA. A preliminary PXRD acquisition to check the purity and crystallinity of the sample was carried out in the 2θ range 2.0–35.0°, with steps of 0.02° and time per step of 1 s. The PXRD acquisition for the crystal structure assessment was then performed overnight in the 2θ range 2.0–105.0°, with steps of 0.02° and an overall scan time of about 12 h. As witnessed by a visual comparison among the PXRD patterns, **NU-1000-PhTz** shares the same 3D architecture of **NU-1000**(12) and of other already known **NU-1000-FG** MOFs.^31,32^ This suggestion was confirmed by performing an independent indexing procedure consisting in a standard peak search, allowing for the estimation of the first 20 low-to-medium angle peak maximum positions that were then processed with the software TOPAS-R V3.0^33^ through the singular value decomposition algorithm,^34^ yielding approximate unit cell parameters. The space group was assigned on the basis of the observed systematic absences. The crystallographically independent portion of the pyrene-based ligand and the thiazolium-based ligand was described using rigid bodies built up through the *z*-matrix formalism, assigning average values to bond distances and angles.^35^ In the initial steps of the structure determination, both the metal cluster constituents (i.e., Zr^4+^, O^2–^, H_2_O, and OH^–^) and the pyrene-based ligand were positioned according to the crystal structure of NU-1000-NDC (H_2_NDC = naphthalene-2,6-dicarboxylic acid).^32^ The thiazolium-based ligand, the bromide anion, and a number of oxygen atoms with variable site occupancy factor modeling smeared electron density in the triangular channels and in the cavities containing **PhTz**^**–**^ were located using the simulated annealing approach^36^ implemented in TOPAS-R V3. During the structure refinement stages, carried out with the Rietveld method, rotations about the single bonds of the pyrene-based and the thiazolium-based ligands were allowed, and the position of the metal cluster constituents was refined according to the symmetry constraints. The background was modeled by using a Chebyshev-type polynomial function. A unique isotropic thermal factor [B_iso_(M)] was refined for the Zr^IV^ ions; the isotropic thermal factor of the other atoms was calculated as B_iso_(L) = B_iso_(M) + 2.0 (Å^2^). The peak profile was modeled through the fundamental parameters approach.^37^ The final Rietveld refinement plot is shown in Figure S4 of the Supporting Information.

Crystallographic data for **NU-1000-PhTz**: hexagonal, *P*6/*mmm*, *a* = 39.602(2) Å, *c* = 16.440(1) Å, *V* = 22 329(2) Å^3^, *Z* = 24, *Z*’ = 3, ρ = 0.567 g cm^–3^, *F*(000) = 3771.8, *R*_Bragg_ = 0.014, *R*_p_ = 0.053, and *R*_wp_ = 0.075, for 5151 data and 45 parameters in the 2.0–105.0° (2θ) range. CCDC no. 2085493.

### Variable-Temperature PXRD

The thermal behavior of **NU-1000-PhTz** was studied *in situ* by means of variable-temperature PXRD, depositing a powdered sample (∼20 mg) on an aluminum sample holder and heating it through a custom-made sample heater (Officina Elettrotecnica di Tenno, Ponte Arche, Italy) in the temperature range 303–763 K, with steps of 20 K. A PXRD pattern was acquired under isothermal conditions at each step, in the 2θ range 4.0–20.0°, with steps of 0.02° and a time per step of 1 s. A parametric whole powder pattern refinement carried out with the Le Bail approach allowed to unveil the relative variations of the unit cell parameters in the investigated thermal range.

### *Ex Situ* Heating under N_2_ Flow

To retrieve information about the chemical identity of the solid residue after thermal decomposition, ∼20 mg of **NU-1000-PhTz** was placed in an oven and heated at 1023 K for 15 min under N_2_ flow. After cooling down to room temperature, a PXRD pattern was acquired with the Bruker AXS diffractometer described above in the 2θ range 5.0–105.0°, with steps of 0.02° and a time per step of 1 s. A qualitative analysis was carried out based on the Powder Diffraction File database release 2001 (ICDD—International Centre for Diffraction Data) and confirmed by means of a whole powder pattern refinement carried out with the Le Bail method.

### Gas Adsorption

**NU-1000-PhTz** (∼40 mg) was activated at 393 K under a high vacuum (10^–6^ Torr) for 12 h before each measurement. The textural properties were evaluated through volumetric N_2_ adsorption isotherms recorded at 77 K on an ASAP 2020 Micromeritics instrument. For the Brunauer–Emmett–Teller (BET) specific surface area calculation, the 0.01–0.1 *p*/*p*^0^ pressure range of the isotherm was used to fit the data. Within this range, all the Rouquerol consistency criteria are satisfied.^38,39^ The total pore volume was estimated at *p*/*p*^0^ = 0.98. The micro- and mesopore sizes were evaluated through NLDFT methods (Tarazona model for cylindrical pores). CO_2_ and N_2_O adsorption isotherms were recorded at 213, 253, 273, 298, 313, and 323 K at a maximum pressure of 1.2 bar. The isosteric heat of adsorption (*Q*_st_) values of both gases were calculated from the six isotherms according to the differential form of the Clausius–Clapeyron equation:^40,41^
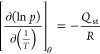
1where *R* is the gas constant (8.314 J K^–1^ mol^–1^). The IAST A/B adsorption selectivity (A, B = CO_2_, N_2_O, or N_2_) of binary mixtures at a total pressure of 1 bar^42^ and at *T* = 298 and 323 K was determined as the ratio of the adsorbed molar fractions of the two gases divided by the ratio of the gas-phase initial molar fractions:^43^
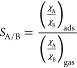
2

The (χ_A_)_ads_ and (χ_B_)_ads_ values were derived from the application of the free software pyIAST (https://github.com/CorySimon/pyIAST) to the experimental single-component isotherms collected at the chosen temperature. The initial compositions (%) for the calculation were the following: [15:85] for the [CO_2_/N_2_] and [N_2_O/N_2_] pairs and [50:50] for the [N_2_O/CO_2_] pair. These ratios were selected to mimic the general feed composition of the landfill and flue gases, respectively.^44^ The Henry model was employed for the isotherm fitting. For a detailed explanation of these models and the related parameters, see the pyIAST Web page and documentation.

### Computational Details

The adsorption of CO_2_ and N_2_O was simulated by Grand Canonical Monte Carlo (GCMC) methods using the RASPA software package.^45^ The framework was assumed to be rigid (i.e., atoms were frozen in the position assessed by crystal structure determination), and part of the point charges of the framework were distributed according to the QEq method using the code of Wells et al.^46^ Dispersive and electrostatic interactions between the framework and the adsorbed molecules were taken into consideration during the simulations. The Lennard-Jones (LJ) equation was used to describe dispersive interactions, and its parameters were calculated by the Lorentz–Berthelot mixed rule. For the framework, the LJ parameters of the metal atoms were taken from the UFF force field,^47^ while those of the other elements were taken from the DREIDING force field.^48^ This combination of force field parameters has already been successfully used to simulate gas adsorption in porous materials.^49,50^ A rigid three-point charged LJ linear model was used for CO_2_ and N_2_O. The energy parameters of CO_2_ were taken from the EPM2 force field,^51^ and the C–O bond length was set at 1.149 Å. The energy parameters of N_2_O were adopted from the work of Chen et al.,^52^ with the N–N and N–O bond lengths of 1.1282 and 1.1842 Å, respectively. The number of MOF unit cells in the simulation box was 1 × 1 × 2 to ensure that the simulation unit was extended to be at least 28.0 Å along each dimension. Periodic boundary conditions were applied. The dispersive interactions were calculated using a long-range correction with a spherical cutoff radius of 14.0 Å, while the Ewald sum was used to consider the electrostatic interactions. The Peng–Robinson equation of state was used to convert the fugacity. 50 000 cycles of simulations were performed, including 25 000 equilibrium cycles and 25 000 ensemble average cycles. In each cycle, the adsorbed molecules underwent three types of trials: translation, rotation, and regeneration. Further increasing the number of cycles had no significant effect on the adsorption results. Molecular dynamics (MD) simulations were performed according to the experimental conditions. One molecule was inserted into the unit cell of each MOF using a canonical (*NVT*) ensemble to study the diffusion behavior of CO_2_ and N_2_O. Constant temperature conditions were maintained using a Nosé–Hoover chain (NHC) thermostat.^53^ The velocity Verlet algorithm was used to integrate Newton’s equation of motion. The simulation steps and the time per step of each MD simulation were 6 ns cycles and 1 fs, respectively, preceded by an equilibration of 3 ns. Finally, the slope of the molecular mean-square displacement (MSD) versus time plot (in its initial time interval, where a satisfactorily linear trend can be observed) was used to calculate the molecular self-diffusion coefficient (*D*_s_), averaging over 10 independent trajectories.^54,55^

## Results and Discussion

### Synthesis and Solid-State Characterization of **NU-1000-PhTz**

Thiazolium bromide dicarboxylic acid (**H**_**2**_**PhTz**)Br is prepared through a simple thiazole *N*-quaternization reaction starting from the commercially available thiazole-5-carboxylic acid and 4-bromomethyl benzoic acid (Scheme 1). The salt is sparingly soluble in acetonitrile; it precipitates out of the solution mixture in pure form and high yield (84%). Expectedly for amino acids, (**H**_**2**_**PhTz**)Br crystallizes from methanol/acetonitrile in its *zwitterionic* neutral form (**HPhTz**) after HBr elimination (Scheme S1).

**Figure 2:**
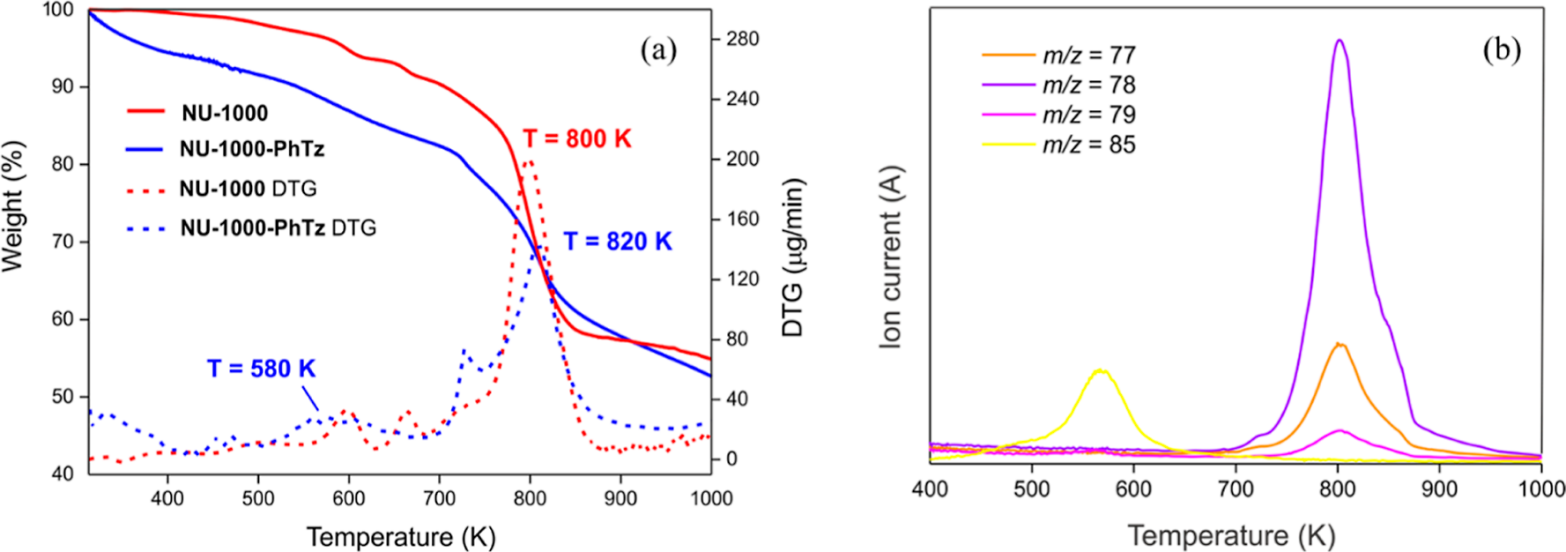
(a) Comparison of TGA-DTG profiles of **NU-1000** and **NU-1000-PhTz**. (b) Characteristic mass peaks for thiazole (*m*/*z* = 85 amu) and phenyl (*m*/*z* = 77, 78, and 79 amu) as a function of temperature during the thermal decomposition of **NU-1000-PhTz**.

In the crystal structure of **HPhTz**, the thiazole carboxylic group is deprotonated (−COO^–^), while the benzoic moiety is in its protonated (−COOH) form. The carboxylic–carboxylate hydrogen-bonding interactions, combined with the π–π stacking of the aromatic rings, generate dimers (Figure S2). These dimeric units are further assembled through an intricate net of hydrogen-bonding interactions involving the oxygen atoms of the carboxylic and carboxylate groups, the sulfur atoms, and the two crystallization water molecules, generating a 3D supramolecular architecture. Inclusion of (**H**_**2**_**PhTz**)Br into **NU-1000** was achieved following the same experimental conditions successfully employed for a similar benzothiazolium monocarboxylate prepared by us at the beginning of 2020.^23^**NU-1000-PhTz** has been thoroughly characterized in the solid state. The IR spectroscopic analysis cannot undoubtedly confirm the extra ligand insertion, the main vibrational modes being almost identical for **NU-1000** and **NU-1000-PhTz** in the 2000–400 cm^–1^ wavenumber range (Figure S5). However, comparison of the difference [(**NU-1000-PhTz**) – (**NU-1000**)] spectrum with that of pure (**H**_**2**_**PhTz**)Br (Figure S6) highlighted some typical bands of the latter at 1663 cm^–1^ [ν(COO)], 1612 cm^–1^ [ν(C=C)], 1419 cm^–1^ [δ(CH_2_)], and 768 cm^–1^ [γ(CH)]. The XRF qualitative analysis of **NU-1000-PhTz** (Figure S7) highlighted the presence of sulfur and bromine, confirming the successful SALI functionalization and revealing that the (**H**_**2**_**PhTz**)Br ligand is incorporated within the MOF in its doubly deprotonated (**PhTz**^–^) and not zwitterionic (**HPhTz**) form; the bromide anion is then necessary to balance the overall framework charge. PXRD preliminarily suggested that the parent crystallographic symmetry and network structural motif remain unaltered after functionalization; as expected, differences in the relative intensities of the diffraction peaks were observed, due to the changes in the electron density distribution introduced by **PhTz**^–^ within the unit cell. **NU-1000-PhTz** crystallizes in the hexagonal space group *P*6/*mmm*. The inorganic secondary building unit is an oxo–hydroxo cluster made of six octahedrally coordinated Zr^IV^ cations connected to four μ_3_-O^2–^ and four μ_3_-OH^–^ anions (Figure 1a). The NU-1000 skeleton is built through the coordination of each [Zr_6_] metallic node to eight different carboxylates coming from TBAPy^4–^. As preliminarily verified by describing the electronic density not belonging to the framework with dummy atoms (Figure S8), mimicking what was previously done for NU-1000-NDC,^32^ the ∼8 Å cavities that lie along the *c*-axis are occupied by the bridging thiazolium salt (Figure 1b), bonded to the [Zr_6_] nodes through its carboxylate groups (Zr–O distance in the 2.027(5)–2.433(9) Å range). The observed distribution of the extra framework electronic density excludes the fact that the pillar is clathrated within the micro- or mesoporous cavities. The presence of the pillar in a mono-deprotonated zwitterionic and bromine-free form (**HPhTz**, Scheme S1) can also be excluded, as bromine in **NU-1000-PhTz** was directly detected through X-ray fluorescence and X-ray photoelectron spectroscopy. The position of the pillar leads to a MOF possessing the rare **{4,10}-c** network with the topological point symbol {3^2^·4^2^·5^2^}_2_{3^8^·4^16^·5^8^·6^13^} (Figure S9).^56^ To the best of our knowledge, the same topology is shown only by NU-1000-NDC,^32^ F-BA-NU-1000 (BA = benzoate),^31^ PCN-608-NH_2_-BDC (NH_2_-H_2_BDC = 2-aminoterephthalic acid),^57^ and PCN-608-SBDC (H_2_SBDC = 2-sulfoterephthalic acid).^57^ The loading of one **PhTz**^**–**^ ligand per [Zr_6_] node was confirmed. Thus, based on the ligands relative stoichiometric ratio, the MOF minimal formula can be written as [Zr_6_O_4_(OH)_6_(H_2_O)_2_(TBAPy)_2_(**PhTz**)]Br. The remaining free coordination sites of the [Zr_6_] cluster in **NU-1000-PhTz** are saturated by four hydroxide/aquo ligands oriented toward the 3 nm wide hexagonal pores and interacting with the Br^–^ anions coming from the added extra linker (distance O···Br, 2.79(6) Å) (Figure 1c). The presence of an O–H···Br hydrogen-bond interaction is also witnessed by the shift at higher binding energies of the Br 3d XPS spin–orbit peaks 3d^3/2^ and 3d^5/2^ when passing from (**H**_**2**_**PhTz**)Br (68.4 and 67.5 eV) to **NU-1000-PhTz** (69.7 and 68.6 eV, Figures S10 and S11). This clearly indicates a reduction of electron density of the bromide ion when included within the MOF mesopores. The same kind of shift has been recently observed in MOF-5^58^ or HKUST-1^59^ loaded with the ionic liquid 1-butyl-3-methylimidazolium bromide (BMIMBr), as a consequence of the Br^–^···M^II^ interaction (M = Zn, Cu). The intensity of the first PXRD peak at 2θ ≈ 2.5° is not appreciably affected by the extra linker addition, at variance with what was observed in other SALIed **NU-1000** MOFs like Ru(bpy)_2_(dcbpy)@**NU-1000** (bpy = 2,2′-bipyridine; dcpby = 4,4′-dicarboxy-2,2′-bipyridine),^13^ or H_3_PW_12_O_40_@**NU-1000**.^14^ This proves that in the case of **NU-1000-PhTz** the functionalization involves the mesopores only marginally (through the Br^–^ ions located at the edges of the hexagonal channels). The location of the framework counterions in the hexagonal channels was already observed in the “PCN-608-FG” MOF family.^57^ The linker loading and bridging coordination mode are the same as that observed for other dicarboxylic acids SALIed to [Zr_6_] nodes like NU-1000-NDC^32^ and NU-901-NDC^60^ or longer analogues in the 8-connected zirconium MOF PCN700.^61^ The node-to-node distance along the *c*-axis in **NU-1000** (∼8.5 Å) is comparable to the carboxylate-to-carboxylate distance measured in free **HPhTz** (in the range ∼8.5–11.5 Å for the two independent molecules). The flexible nature of **PhTz**^**–**^ induced by the methylenic −CH_2_– bridge connecting the two aromatic rings together with a certain framework flexibility shown by the NU-1000-type architecture (Table S2) allowed for the successful insertion of the extra ligand in such a narrow space. Smeared residual electron density was detected in both the triangular cavities and the ∼8 Å cavities and modeled using oxygen atoms for the sake of simplicity. Neglecting the smeared electron density, the empty volume estimated with the software PLATON^62^ is ∼69%, which is lower than that of **NU-1000** and NU-1000-NDC-HCl (showing a bridging linear pillar in the triangular channels) but comparable to that of F-BA-NU-1000, where the extra ligands dangle from the [Zr_6_] nodes in the triangular cavity (Table S2). At odds with what was observed with NU-1000-BzTz,^23^ TGA (Figure 2a) showed that the thermal stability of **NU-1000-PhTz** is slightly higher than that of **NU-1000** (*T*_dec_ = 820 vs. 800 K, respectively). An initial weight loss of *ca.* 19 wt % (in line with the stoichiometric 1:1 [Zr_6_]/**PhTz**^**–**^ ratio) can be reasonably ascribed to **PhTz**^**–**^ decomposition. Indeed, the DTG peak found in this range falls at *T* = 580 K, a value that is close to that found for the decomposition of isolated (**H**_**2**_**PhTz**)Br, occurring at *T* = 545 K (Figure S12). Further proof of evidence is provided by the MS analysis of the volatiles (Figure 2b), where a peak at *m*/*z* = 85 amu, typical of thiazole, appears in the same temperature range. MOF decomposition at 820 K is witnessed by the presence in the MS spectra of the volatiles of peaks at *m*/*z* = 77, 78, and 79 amu, typical of phenyl rings. After the decomposition, nanocrystalline ZrO_2_ is formed, as unveiled by the PXRD pattern of the solid recovered after heating *ex situ* at 1023 K for 15 min under N_2_ flow (Figure S13).

**Figure 3:**
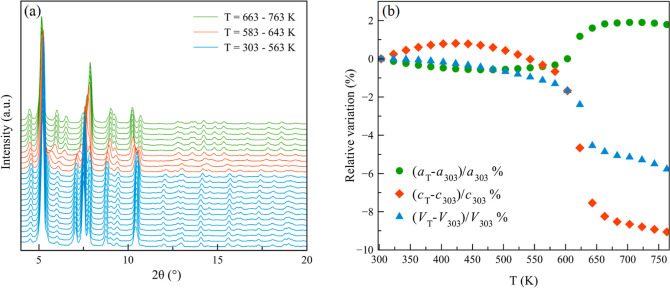
(a) Variable-temperature PXRD patterns of **NU-1000-PhTz** acquired in air, with steps of 20 K, in the temperature range 303–763 K; in red, PXRD patterns showing the highest peak shift (and *c*-axis variation). (b) Percentage relative variation of the unit cell parameters as a function of the temperature.

The variable-temperature PXRD experiment carried out on **NU-1000-PhTz** evidenced that the material maintains its crystallinity at least up to 763 K, as depicted in Figure 3a. In the 303–583 K temperature range, the unit cell parameters variation is less than 1.4% (volumetric thermal expansion coefficient α_V_ ∼ −0.2 × 10^–6^ K^–1^), which is evidence of the structural rigidity of the MOF in this temperature range. Starting from ∼583 K, a significant decrease of the *c*-axis is observed (−6.9% in the temperature range 583–643 K; linear thermal expansion coefficient, α_c_ ∼ −2.4 × 10^–6^ K^–1^) (Figure 3b) and tentatively associated to **PhTz**^**–**^ loss, as highlighted by TGA and MS (*vide supra*). Indeed, by applying the so-called Kempster–Lipson rule^63^ that assigns to each nonhydrogen atom a volume of ∼18 Å^3^, the volume occupied by **PhTz**^**–**^ amounts to ∼324 Å^3^, which is consistent with the volume shrinkage of 4.5% estimated in the temperature range 303–643 K (resulting in a decrease of ∼336 Å^3^ per formula unit). Worthy of note, the notable shrinkage of the *c*-axis upon **PhTz**^**–**^ loss is an additional proof of the existence and location of a tetra-coordinated pillar as a bridge between two nodes in the ∼8 Å cavities.

**Figure 4:**
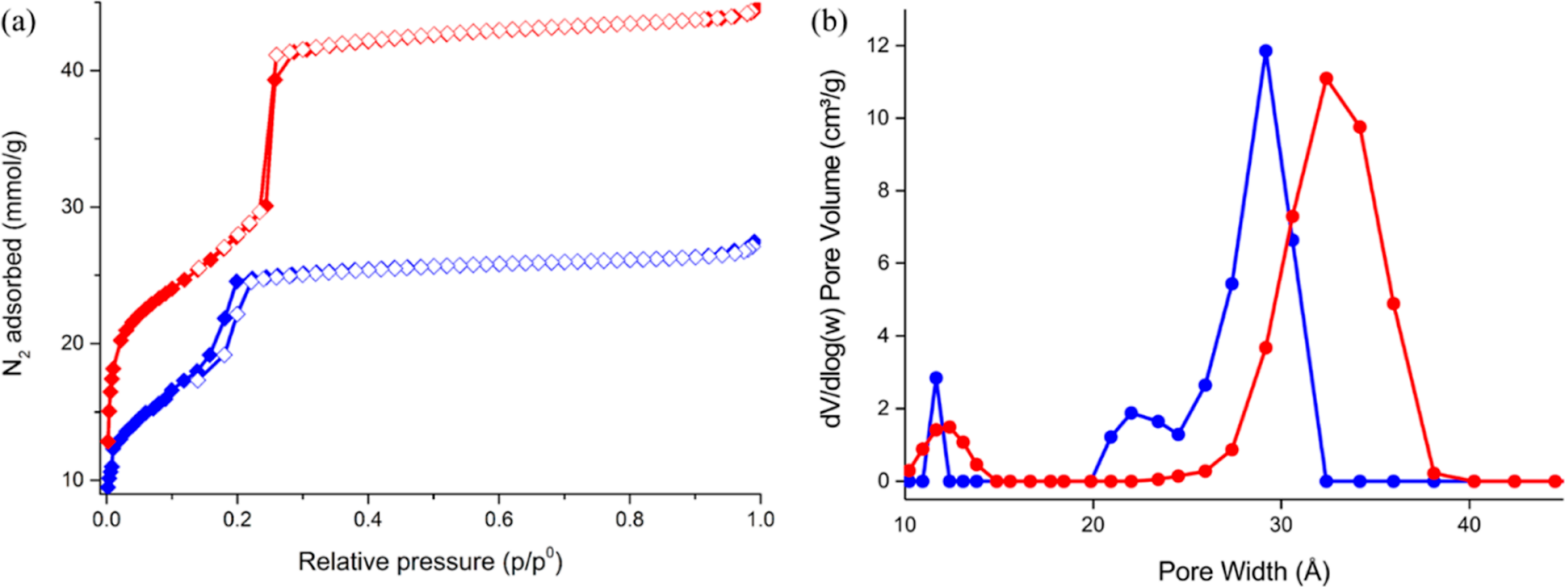
(a) Comparison of N_2_ adsorption isotherms of **NU-1000** (red diamonds) and **NU-1000-PhTz** (blue diamonds). The desorption branch is depicted with empty symbols. (b) Comparison of NLDFT (Tarazona model for cylindrical pores) pore size distribution plots for **NU-1000** (red circles) and **NU-1000-PhTz** (blue circles).

The porosity of **NU-1000-PhTz** was evaluated through volumetric N_2_ adsorption at 77 K on preactivated samples (Figure 4a). The isotherm shape of Type IV is the same as that of **NU-1000**, but the mesopore step typical of this MOF family is smaller than that found in **NU-1000**. This is an additional proof of evidence of the partial mesopore filling. The BET surface area is lower than that of pristine **NU-1000** (1560 vs. 2140 m^2^/g, respectively), with a total pore volume of 0.93 versus 1.53 (**NU-1000**) cm^3^/g. The same behavior was observed in NU-1000-BzTz, with a monodentate dangling group protruding into the **NU-1000** mesopores.^23^ Likewise, the BET specific surface area of NU-1000-NDC, with excess NDC mono-grafted linkers dangling into the mesopores, is 1720 m^2^/g, versus 2030 m^2^/g of NU-1000-NDC-HCl, where the mono-grafted linkers were removed by HCl_(aq)_ treatment.^32^ Analogously, for the R-BA-NU-1000 series (R = −NH_2_, −OCH_3_, −CH_3_, −H, −F, and −NO_2_) with mono-grafted *para*-R-benzoate linkers dangling into the microporous channels, the BET SSA ranges from 1660 to 1900 m^2^/g.^31^ Despite the thiazolium pillar insertion in the microporous cavities, the micropore size (Figure 4b) remains practically unchanged when passing from **NU-1000** (12.4 Å) to **NU-1000-PhTz** (11.6 Å). This behavior was already observed in NU-1000-NDC-HCl^32^ and in the R-BA-NU-1000 series. On the other hand, the mesopores are smaller in **NU-1000-PhTz**, passing from *w* = 33 to 29 Å, respectively (Figure 4b). This is not unexpected, given the presence of the bromide counter ions in the hexagonal mesopores. In addition, the mesopore step occurs at a lower relative pressure than in **NU-1000**. All these data taken together prove that SALI involves both micropores and (to a lesser extent) mesopores, at odds with what was found for NU-1000-BzTz,^23^ NU-1000-NDC-HCl,^32^ and some other NU-1000-FG derivatives of the literature.^11,15,20^

### CO_2_ and N_2_O Adsorption on **NU-1000-PhTz**

**Figure 5:**
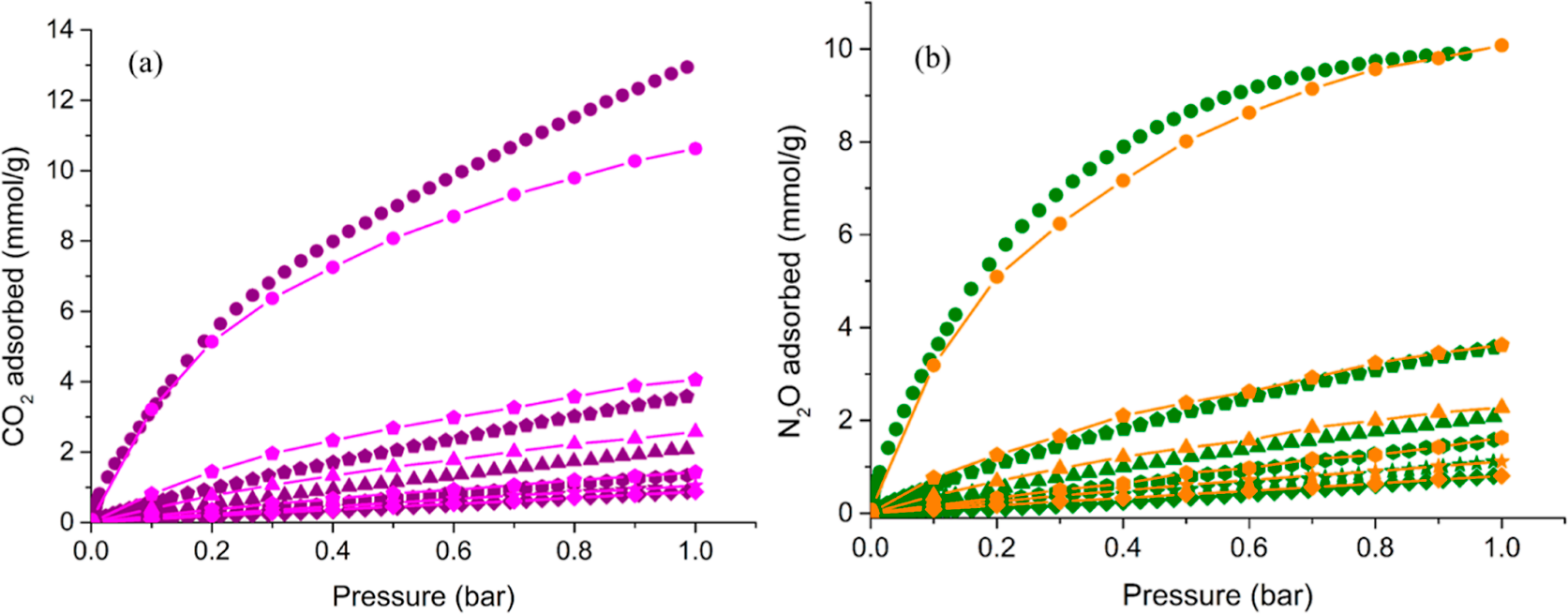
Comparison between experimental (purple and green symbols) and simulated (magenta and orange lines + symbols) CO_2_ (a) and N_2_O (b) adsorption isotherms of **NU-1000-PhTz** at *T* = 323 K (diamonds), 313 K (stars), 298 K (hexagons), 273 K (triangles), 253 K (pentagons), and 213 K (dots).

The activated material has been tested in CO_2_ and N_2_O adsorption at *p*_max_ = 1.2 bar and at variable temperatures between *T* = 213 and 298 K. As found in NU-1000-BzTz,^23^**NU-1000-PhTz** showed an increased affinity for carbon dioxide when compared with its parent MOF. The total CO_2_ uptake at *p*_CO_2__ = 1 bar and *T* = 298 and 273 K is 6.2 wt % (1.4 mmol/g) and 9.5 wt % (2.2 mmol/g), respectively (Figure 5a). The absolute gas uptake at ambient temperature is comparable to that found for other thiazole-containing MOFs like NU-1000-BzTz (8.7 wt %),^23^ Zr_6_(O)_4_(OH)_4_(TzTz)_6_ (7.5 wt %, TzTz^2–^ = [2,2′-bithiazole]-5,5′-dicarboxylate),^24^ or Cu(5-Tz)_2_ (9.0 wt %, 5-Tz^–^ = thiazole-5-carboxylate),^27^ but it is half of that measured for **NU-1000** (2.8 mmol/g at 298 K)^15^ because of the lower specific surface area. In terms of CO_2_ isosteric heat of adsorption at zero coverage (*Q*_st_), the thiazolium-functionalized MOF is featured by a higher *Q*_st_ value than that found for its parent analogue (25 vs. 17^15^ kJ/mol, respectively, Figure S14). This value is identical to that found in NU-1000-BzTz (as expected for a similar pore decoration),^23^ and it falls in the range calculated for other perfluoroalkane-functionalized^15^ or peptide-functionalized^20^**NU-1000** samples studied in the literature (between 24 and 34 kJ/mol). The isosteric heat of adsorption reflects the thermodynamic affinity of the material for CO_2_; the introduction of a polar molecule like a thiazolium salt into the MOF channels is beneficial for the MOF–CO_2_ interaction. Screening for good adsorbents of other polluting gases, **NU-1000-PhTz** has also been tested as a nitrous oxide sponge under the same pressure and temperature conditions used for carbon dioxide. The total N_2_O uptake at *p*_N_2_O_ = 1 bar and *T* = 298 and 273 K is 7.2 wt % (1.6 mmol/g) and 9.4 wt % (2.1 mmol/g), respectively (Figure 5b). These values are lower than those found in the Ni-based MOF [Ni(bptc)_0.5_(H_2_O)] (12.4 wt % at 298 K; bptc^4–^ = biphenyl-3,3′,5,5′-tetracarboxylate)^64^ but higher than those measured in MOF-5 (≈4.0 wt %).^65^ The N_2_O isosteric heat of adsorption at zero coverage equals 27 kJ/mol (Figure S15), and it is slightly higher than that of CO_2_. This value is higher than that found for [Ni(bptc)_0.5_(H_2_O)] (26.6 kJ/mol)^64^ or for the Zn-based MOFs MFU-4*l* (17.9 kJ/mol) or Li-MFU-4*l* (23.6 kJ/mol).^66^ In addition to the presence of a slightly higher thermodynamic affinity of **NU-1000-PhTz** for N_2_O than for CO_2_ (the first ever reported example of this kind, to the best of our knowledge), an unexpected temperature-dependent preferential adsorption has been found. While at *T* = 298 and 313 K (*p* = 1 atm) the N_2_O uptake is higher than that of CO_2_, the opposite occurs at temperatures falling out of the 298–313 K range. Table 1 lists the adsorption data for CO_2_ and N_2_O for the MOF at various temperatures. This behavior is unprecedented, also given the absence of a comparative study of this kind in the literature. Therefore, **NU-1000-PhTz** may represent a “smart material” for the discrimination of chemically similar polluting gases, opening new horizons in the field of molecular recognition and gas mixture separation. To shed further light on the title MOF adsorption behavior in this context, IAST selectivity (*S*_A/B_) data for [CO_2_/N_2_], [N_2_O/N_2_], and [N_2_O/CO_2_] binary mixtures at two different temperatures (298 and 323 K) were estimated; the results are summarized in Table 2. *S*_N_2_O/CO2_ for an equimolar mixture reaches its maximum value at *T* = 298 K (1.1). This value is higher than that measured for **NU-1000** (0.8) under the same experimental conditions, proving the beneficial effect of the introduction of the thiazolium pillar on the selectivity for N_2_O at 298 K. According to these results, binary N_2_O/CO_2_ equimolar mixtures may be enriched in either component simply through a temperature switch, namely, richer in CO_2_ at 298 K or richer in N_2_O at 323 K. As far as *S*_CO_2_/N_2__ and *S*_N_2_O/N_2__ are concerned, the absolute values are much higher than those of *S*_N_2_O/CO_2__ because of the nonpolar nature of nitrogen. The absolute values increase as a function of the temperature; the highest values were recorded at *T* = 323 K. At this temperature, the amount of N_2_ adsorbed is close to zero. Therefore, N_2_ separation from both greenhouse gases is more efficient if compared with that achieved at ambient temperature.

**Table 1:** CO_2_ and N_2_O Adsorption Data of Experiments and Simulations at *p* = 1 bar for **NU-1000-PhTz**

	CO_2_ adsorbed [mmol/g]		N_2_O adsorbed [mmol/g]	
temperature [K]	experiments	simulations	experiments	simulations
323	0.9 (4.0 wt %)	0.9 (4.0 wt %)	0.8 (3.6 wt %)	0.8 (3.6 wt %)
313	1.1 (4.7 wt %)	1.0 (4.7 wt%)	1.2 (5.0 wt %)	1.1 (5.0 wt %)
298	1.4 (6.2 wt %)	1.4 (6.2 wt %)	1.6 (7.2 wt %)	1.6 (7.2 wt %)
273	2.2 (9.5 wt %)	2.6 (11.1 wt %)	2.1 (9.4 wt %)	2.3 (10.2 wt %)
253	3.7 (16.2 wt %)	4.0 (17.7 wt %)	3.6 (16.0 wt %)	3.6 (16.0 wt %)
213	13.1 (57.7 wt %)	10.6 (46.8 wt %)	9.9 (43.4 wt %)	10.1 (44.2 wt %)

**Table 2:** IAST Adsorption Selectivity Data of Binary Gas Mixtures for **NU-1000-PhTz**

temperature [K]	CO_2_/N_2_ [15:85]	N_2_O/N_2_ [15:85]	N_2_O/CO_2_ [50:50]
298	12	14	1.1
323	37	32	0.9

### GCMC Simulations of CO_2_ and N_2_O Adsorption Isotherms on **NU-1000-PhTz** and MD Studies on CO_2_ and N_2_O Diffusion

**Figure 6:**
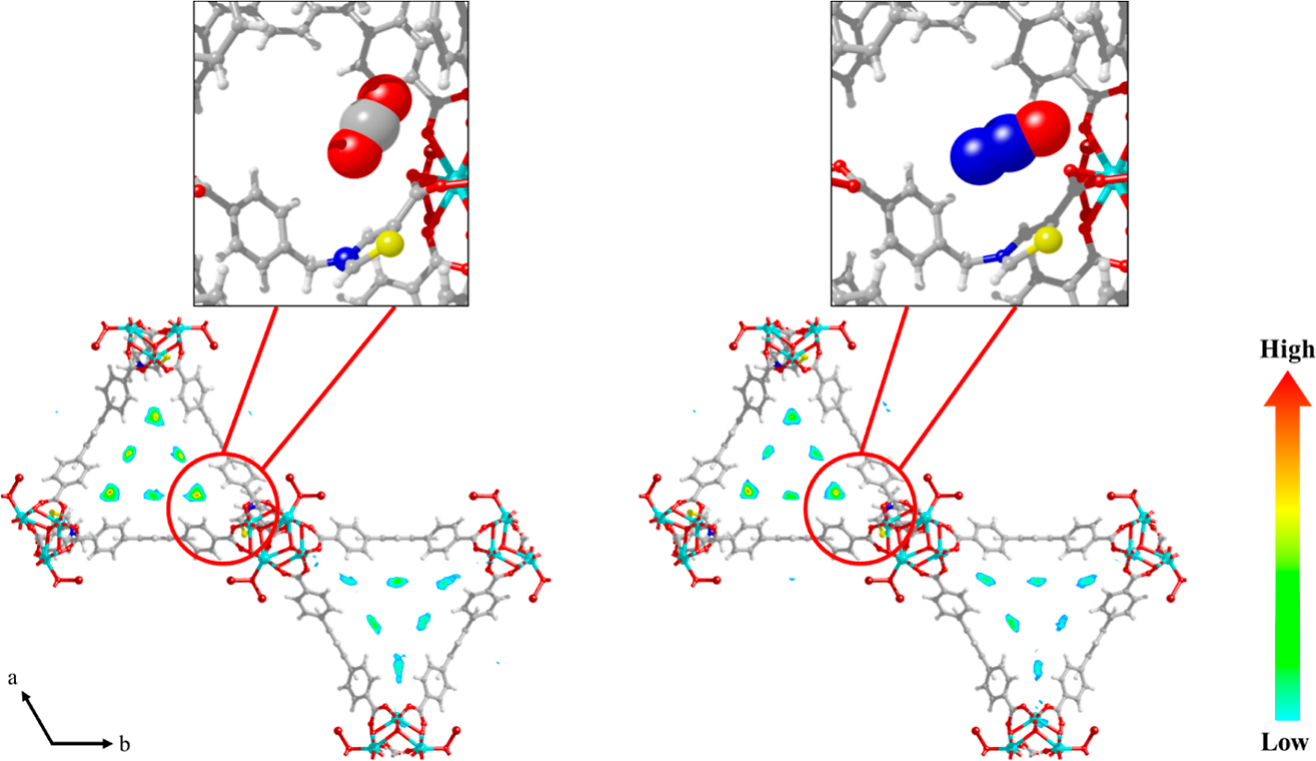
Contour plots of the center-of-mass probability densities of adsorbed CO_2_ and N_2_O in **NU-1000-PhTz** at *T* = 273 K and *p* = 1 bar.

**Figure 7:**
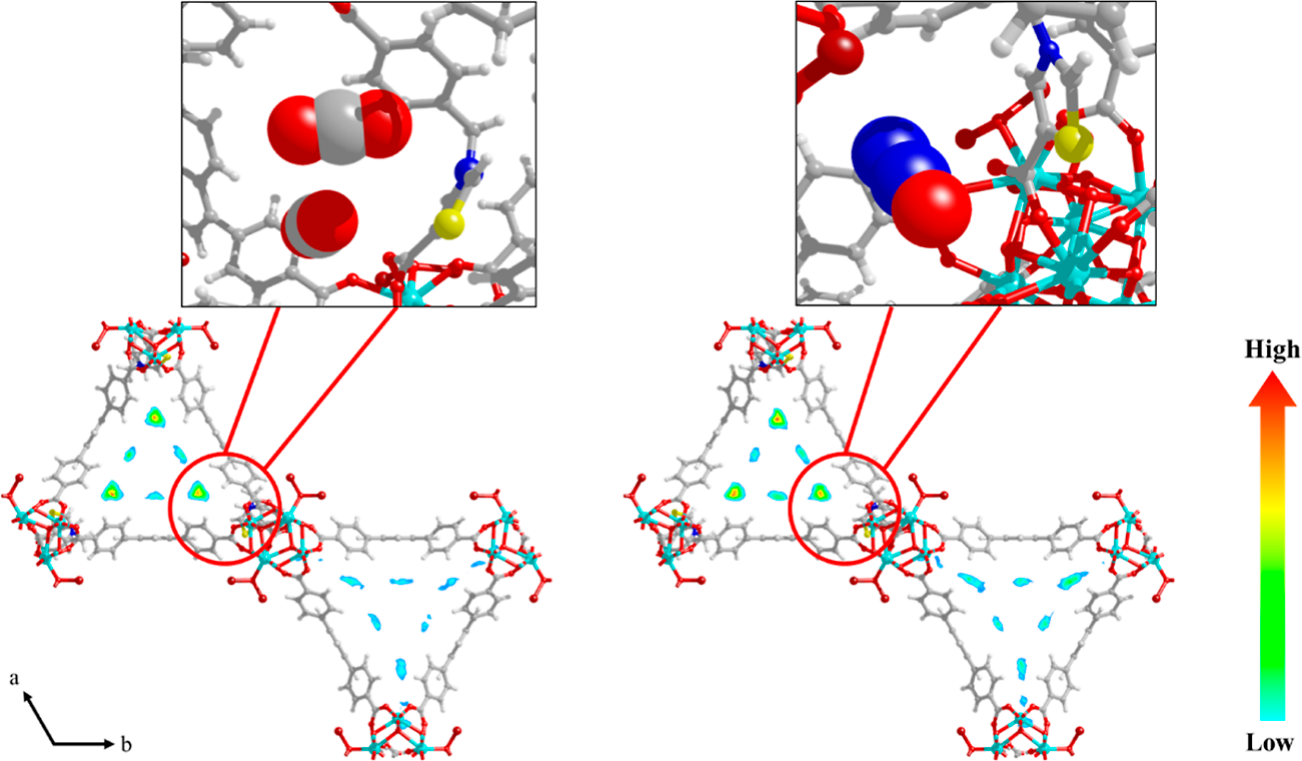
Contour plots of the center-of-mass probability densities of adsorbed CO_2_ and N_2_O in **NU-1000-PhTz** at *T* = 298 K and *p* = 1 bar.

The single-component adsorption isotherms were simulated through GCMC methods and compared with the experimental ones (Figure 5). Previous theoretical calculations of N_2_O in MOFs were carried out at a DFT level of theory on Zn^II^ triazolate frameworks of the MFU-4*l* family (as such or decorated with Li^I^ or Cu^I^ ions)^66^ or on the Co^II^-based Co(BDC)(pz) and Co(BDC)(bpy) MOFs (BDC = terephthalate; pz = 1,4-pyrazine; and bpy = 4,4′-bipyridyl) using an ONIOM model.^67^ The GCMC-calculated adsorbed amounts of CO_2_ and N_2_O obtained through the employment of a mixed UFF/DREIDING force field are in good agreement with the experimental data. In particular, the calculated CO_2_ adsorption capacity is higher than that of N_2_O at 213, 253, 273, and 323 K, while the N_2_O adsorption capacity is higher than that of CO_2_ at 298 and 313 K (Table 1). The contour plots of the center-of-mass probability densities of CO_2_ and N_2_O in **NU-1000-PhTz** at 273 and 298 K along with a zoom of the GCMC snapshots of the most relevant micropore regions are shown in Figures 6 and 7. The preferential adsorption sites of both gases are located at the corners of the triangular channels (micropores), in proximity to the inserted thiazolium pillar. This result confirms the positive effect of the inclusion of a thiazole group on the adsorption of the studied gases, as observed for [Zr_6_(O)_4_(OH)_4_(TzTz)_6_]^24^ or NU-1000-BzTz.^23^ Both guest molecules strongly interact with the framework, but their adsorption modes are different. According to the GCMC snapshots and the derived radial distribution function (RDF) versus interatomic distance [*g*(*r*) versus *r*] plots (Figures S16 and S17 and Table S3), CO_2_ interacts with the thiazolium N and S atoms in an “end-on” (terminal) configuration through its oxygen atoms, revealing a partial positive charge delocalized all over the thiazolium ring. On the other hand, N_2_O prefers a “side-on” configuration where its three atoms give rise to simultaneous interactions with the thiazolium S atom. This different behavior may be ascribed to the polarity of N_2_O, coming from its delocalized charge. The RDF probability maxima reveal that at 298(273) K the shortest N_2_O–framework distances are found at *r* ∼ 3.86(3.90) Å and *r* ∼ 4.06(4.16) Å between the O/S and N^1^/S atoms (N^1^=N^2^=O), respectively. In the case of CO_2_, the shortest contact is between the O/S atoms at r ∼ 4.06(4.06) Å. The S atom of the thiazolium ring is less sterically hindered than the N^+^ atom on the same ring, and this is probably at the origin of its strongest interaction with the guest molecules in the pores. Based on the interaction distances, at both temperatures, both gases preferentially interact through their O atoms (in N_2_O, the negative charge is more likely to be localized on oxygen than on N^1^ nitrogen, for electronegativity reasons). Molecular dynamics studies on the diffusion of the two gases in **NU-1000-PhTz** (Figure S18) have revealed that at all the essayed temperatures below ambient the diffusion coefficient (*D*_s_, Table S4) of CO_2_ is larger than that of N_2_O. However, at 298 K, the diffusion coefficient of N_2_O increases significantly and exceeds that of CO_2_. Therefore, at this temperature, N_2_O preferentially occupies the MOF primary adsorption sites because of its faster diffusion. This is in line with the higher N_2_O adsorption capacity, thermodynamic affinity, and selectivity at 298 K observed experimentally, and it is promising for **NU-1000-PhTz** exploitation in CO_2_/N_2_O mixtures separation. Conversely, for **NU-1000,** the *D*_s_ values calculated for N_2_O are smaller than those of CO_2_ at all the investigated temperatures (Figure S19 and Table S4).

## Conclusions

The SALI methodology has been successfully applied to **NU-1000** for the preparation of the charged framework **NU-1000-PhTz** containing a bridging thiazolium dicarboxylic acid that connects adjacent [Zr_6_] nodes along the crystallographic *c*-axis. The inserted thiazolium pillar improves the (polar) gas uptake capacity of the pristine MOF, showing excellent performance in the adsorption of both CO_2_ and N_2_O, two main greenhouse gases. **NU-1000-PhTz** is featured by a higher thermodynamic affinity for N_2_O than for CO_2_ (the first case reported so far, to the best of our knowledge) and by an unprecedented temperature-dependent preferential adsorption, storing more N_2_O between 298 and 313 K but more CO_2_ at temperatures out of this range. In addition, at *T* = 298 K, **NU-1000-PhTz** shows a higher N_2_O selectivity and a faster diffusion of this gas in its pores. The functionalized MOF can then discriminate between polluting gases through selective adsorption at different temperatures, possibly enriching a CO_2_/N_2_O mixture in either component only through a simple temperature switch. Given the utmost importance of reducing the greenhouse gas concentration in the Earth atmosphere in coming years, it is essential to develop new functional materials with enhanced adsorption properties to be exploited in this context. The introduction of ionic linkers in MOFs, followed by ion-exchange reactions may further tune their adsorption properties and allow for a precise regulation of their micro- and mesopore environments. The current ongoing research activity in our laboratories is focused on the synthesis of other thiazole-based MOFs with high surface area to be tested in the adsorption and catalytic transformation of greenhouse gases.

## Supporting Information Available

Crystal structure of **HPhTz** and related crystallographic details; ligand quantification in **NU-1000-PhTz** through ^1^H NMR signal integration of a digested sample; Rietveld plot for the refinement of the crystal structure of **NU-1000-PhTz**; IR and XRF spectra of **NU-1000-PhTz**; XPS survey scans and Br 3d high-resolution spectra of (**H**_**2**_**PhTz**)Br and **NU-1000-PhTz**; TG-DTG profile of (**H**_**2**_**PhTz**)Br; CO_2_ and N_2_O isosteric heat of adsorption calculations; RDF plots and main interatomic gas–framework distances calculated through GCMC simulations; MDS versus time plots for CO_2_ and N_2_O in **NU-1000-PhTz** and **NU-1000**; self-diffusion coefficients (*D*_s_) of the two gases as a function of temperature (PDF)
Crystallographic data of **H**_**2**_**PhTz** (CIF)
Crystallographic data of **NU-1000-PhTz** (CIF)

## Supplementary Material

am1c21437_si_001.pdf

am1c21437_si_002.cif

am1c21437_si_003.cif
