# Supplementary material for: Temperature-Dependent Nitrous Oxide/Carbon Dioxide Preferential Adsorption in a Thiazolium-Functionalized NU-1000 Metal–Organic Framework
Source: ACS Appl Mater Interfaces. 2021 Dec 2;13(49):58982–93. doi: 10.1021/acsami.1c21437 (PMC9280722; doi:10.1021/acsami.1c21437)
Supplement: Supplementary file 1 — am1c21437_si_001.pdf [file am1c21437_si_001.pdf]

Temperature-dependent nitrous oxide/carbon dioxide preferential adsorption in a thiazolium-functionalized NU-1000 Metal-Organic Framework

Giorgio Mercuri,[a] Marco Moroni,[b] Simona Galli,[b,] Giulia Tuci,[a] Giuliano Giambastiani,[a,c]*

Tongan Yan,[d] Dahuan Liu,[d,] Andrea Rossin[a,]**

[a] *Istituto di Chimica dei Composti Organometallici (ICCOM-CNR), Via Madonna del Piano 10, 50019 Sesto Fiorentino, Italy. E-mail: a.rossin@iccom.cnr.it*

[b] *Dipartimento di Scienza e Alta Tecnologia, Università dell'Insubria, Via Valleggio 11, 22100 Como, Italy. E-mail: simona.galli@uninsubria.it*

[c] *Institute of Chemistry and Processes for Energy, Environment and Health (ICPEES), UMR 7515 CNRS-University of Strasbourg (UdS), 25, rue Becquerel, 67087 Strasbourg Cedex 02, France.*

[d] *State Key Laboratory of Organic-Inorganic Composites, Beijing University of Chemical Technology, Beijing 100029, China. E-mail: liudh@mail.buct.edu.cn*

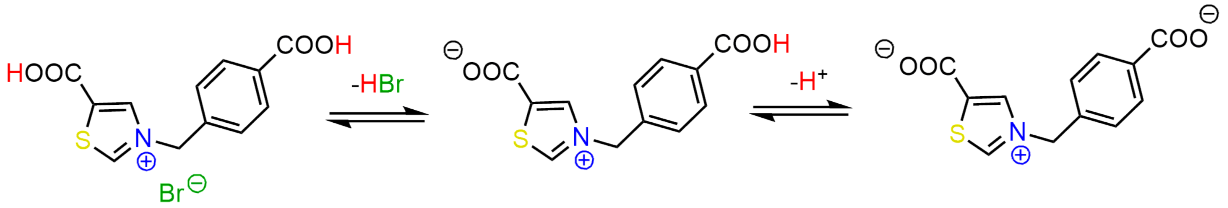

(H_2**PhTz**)Br | **HPhTz** | (**PhTz**)$^-$

Scheme S1. Deprotonation equilibria for (**H$_2$PhTz**)Br.

Single-crystal X-ray Diffraction Structure Determination of HPhTz

Single crystal X-ray diffraction data were collected at T = 100 K on an Oxford Diffraction XcaliburPX diffractometer equipped with a CCD area detector and a sealed X-ray source (Cu Kα, λ = 1.5418 Å). The program used for the data collection was CrysAlis CCD 1.171.[1] Data reduction was carried out with the program CrysAlis RED 1.171,[2] while the absorption correction was applied with the program ABSPACK 1.17. Direct methods as implemented in SIR2014[3] were used to solve the crystal structure, while the structure refinement was performed by full-matrix least-squares against F^2, as implemented in SHELX2018.[4] All the non-hydrogen atoms were refined anisotropically. The hydrogen atoms of the carboxylic groups and of the crystallization water molecule with site occupation factor equal to 1 were located on the difference electron density maps and refined isotropically with U_{iso} = 1.5U_{eq}(O), while all the other hydrogen atoms were fixed in calculated positions (riding model) and refined isotropically with U_{iso} = 1.2U_{eq}(C) (C = carbon atom to which H is bound). As for the water molecule with site occupation factor equal to 0.25, no hydrogen atoms were either located in the electron density map or added in calculated positions. The geometrical calculations were performed by PARST97[5] and molecular plots were produced by the program ORTEP3.[6] CCDC-2085492 contains the supplementary crystallographic data for **HPhTz**. These data can be obtained free of charge from The Cambridge Crystallographic Data Centre *via* https://www.ccdc.cam.ac.uk/structures/. Table S1 collects the main experimental details and crystallographic data.

Table S1. Main crystallographic data and structure refinement details for (**HPhTz**)$_2$·1.25 H_2O.

Formula	$C_{24}H_{20.5}N_2O_{9.25}S_2$
M [g mol^{-1}]	549.04
T [K]	100
Color, habit	Colorless platelets
Size [mm^3]	0.005 × 0.01 × 0.01
Crystal system	Monoclinic
Space group	$P2_1/c$
Z	4
a [Å]	28.383(14)
b [Å]	7.780(5)
c [Å]	10.692(6)
β [°]	91.439(4)
V [Å^3]	2360.3(2)
F(000)	1138.0
μ (Cu Kα) [mm^{-1}]	2.588
D_{calc} [g cm^{-3}]	1.545
θ range for collection [°]	4.7 - 72.3
h, k, l ranges for collection	$-34 \le h \le 33$
	$-9 \le k \le 9$
	$-6 \le l \le 12$
Measured reflns	8500
Unique, observed reflns	4430, 2483
Completeness [%]	98.4
R_{int}, R_σ	0.078, 0.097
Data / restraints / parameters	4430 / 0 / 355
$R1$, w$R2$ [$I > 2\sigma(I)$]	0.061, 0.132
$R1$, w$R2$ (all data)	0.107, 0.170
Gof on F^2	1.091
Max peak, min hole [e Å^{-3}]	0.41, -0.61

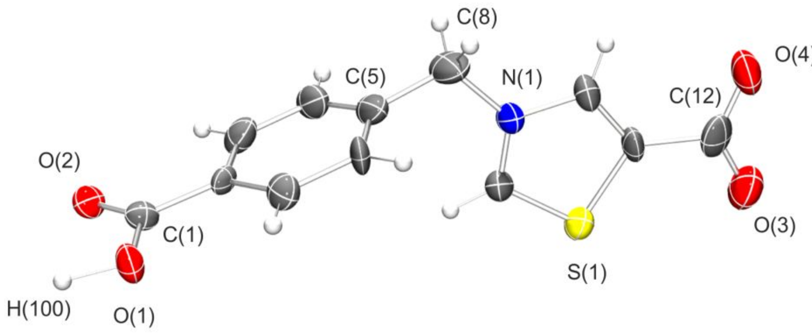

Figure S1. ORTEP drawing of **HPhTz** at 70% probability level. Selected bond lengths (Å) and angles (°): C(1)-O(1) = 1.330(5); C(1)-O(2) = 1.224(5); O(1)-H(100) = 1.01(6); C(12)-O(3) = 1.236(7); C(12)-O(4) = 1.261(7); C(8)-N(1) = 1.477(7); N(1)-C(8)-C(5) = 113.4(4).

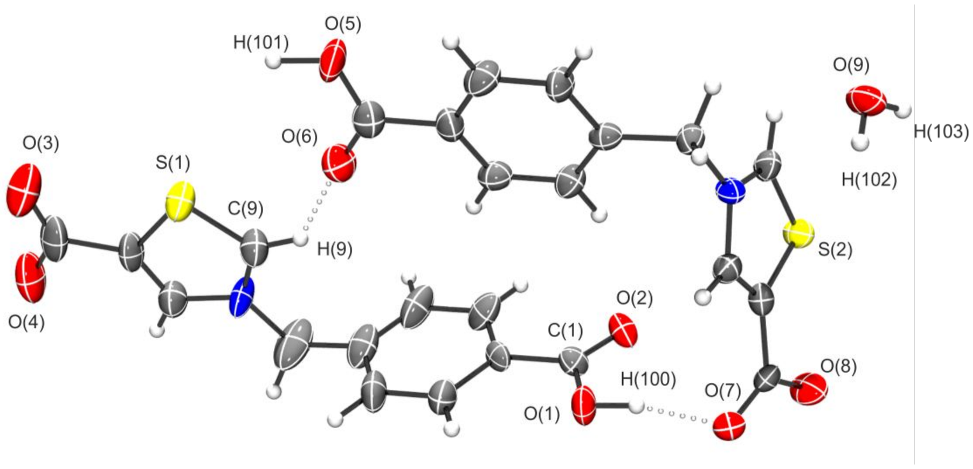

Figure S2. ORTEP drawing of the hydrogen-bonded (**HPhTz**)$_2$ dimer (ellipsoids at 70% probability level). Hydrogen bonds depicted with white dots. Selected D···A distances (Å): C(9)···O(6) = 2.888(6); O(1)···O(7) = 2.646(4); O(5)···O(4)# = 2.503(5); O(9)···S(2)§ = 3.618(4). Symmetry transformations used to generate equivalent atoms: # : -x, y-1/2 ,-z+1/2; § = -x+1, y-1/2, -z+1/2.

Ligand quantification in NU-1000-PhTz

The approximate linkers composition in the MIXMOF has been assessed through ^{1}H NMR analysis of the digested samples in acidic solutions (D_2SO_4 in D_2O, Sigma Aldrich/DMSO-d_6). In a typical procedure, 5 mg of sample was dissolved in three drops of concentrated sulfuric acid and 0.75 mL of DMSO-d_6 directly into an NMR tube. The mixture was heated at T = 363 K for 2 h, to complete the sample digestion. The as-obtained clear yellow solution was analysed *via* ^{1}H NMR (400 MHz, relaxation delay τ = 15 s, 512 scans).

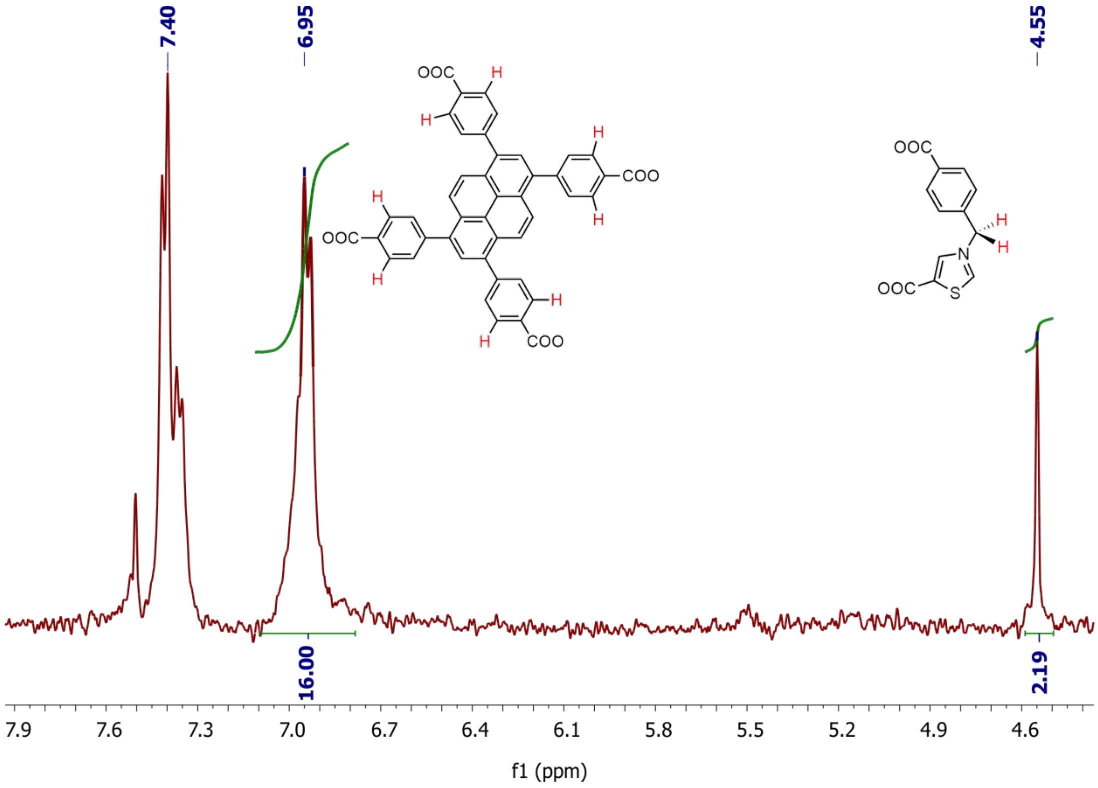

Figure S3. ^{1}H NMR spectrum (400 MHz, D_2SO_4/D_2O/DMSO-d_6, 298 K) of the digested **NU-1000-PhTz** used for the quantification of the functionalization degree.

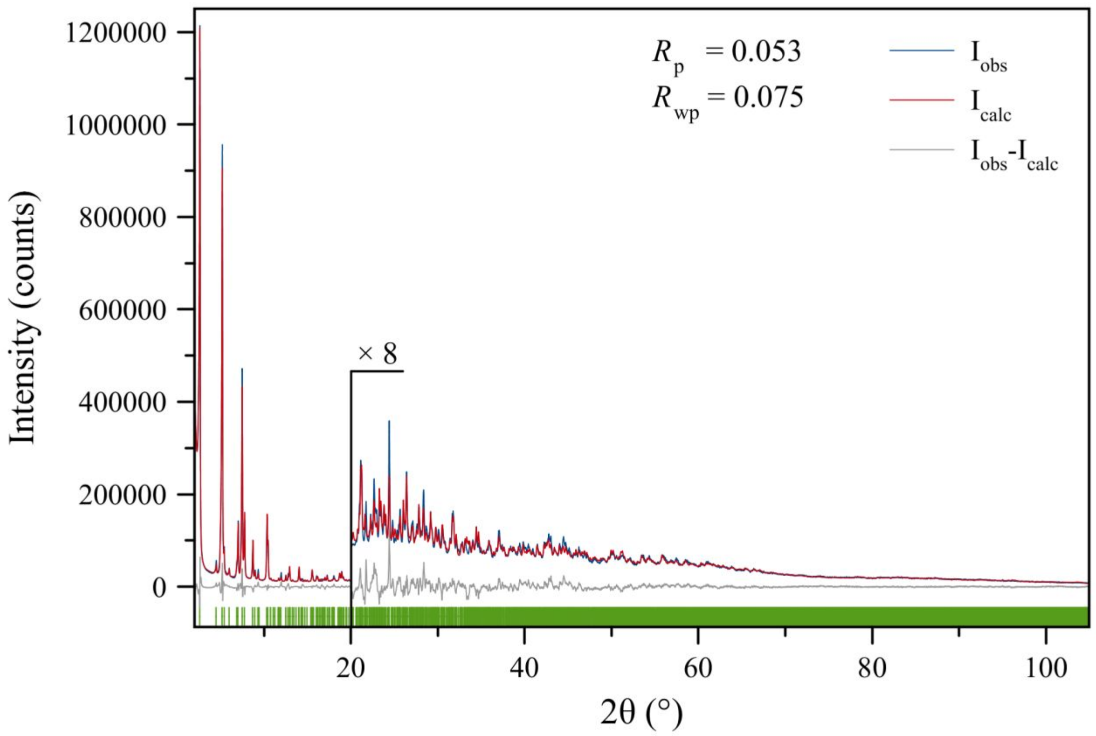

Figure S4. Graphical result of the final structure refinement performed with the Rietveld approach on the PXRD pattern of **NU-1000-PhTz** in terms of experimental, calculated and difference traces (blue, red and grey, respectively). The green markers at the bottom indicate the positions of the Bragg reflections.

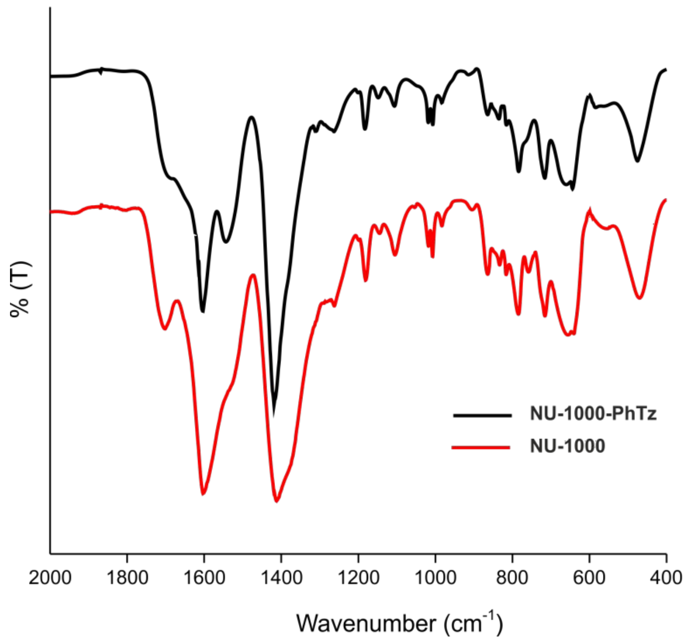

Figure S5. Infrared spectra (KBr, T = 298 K) of **NU-1000** and **NU-1000-PhTz** at comparison.

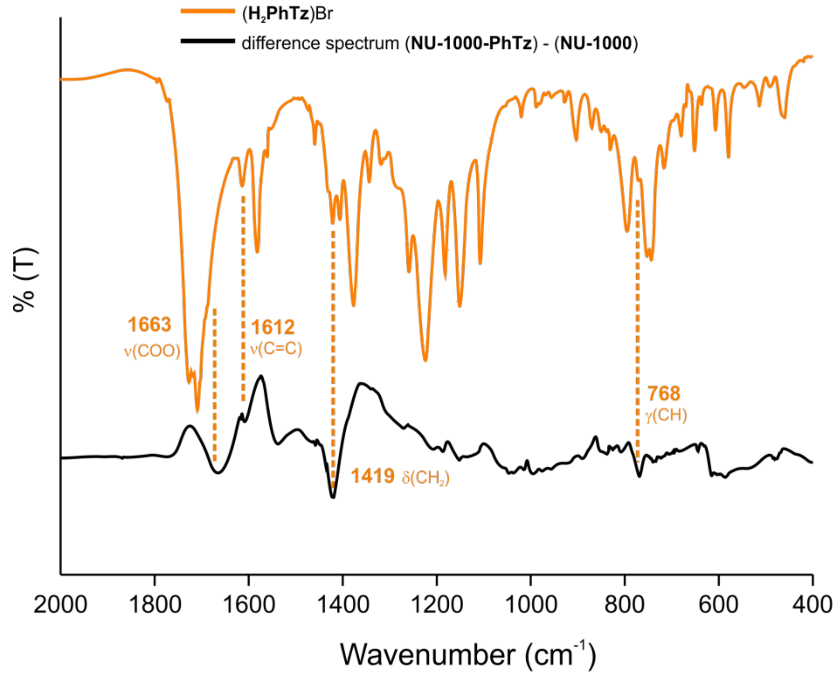

Figure S6. Infrared difference [(**NU-1000-PhTz**)-(**NU-1000**)] spectrum (KBr, T = 298 K, black) and (**H_2PhTz**)Br spectrum (KBr, T = 298 K, orange) at comparison. Some typical thiazolium normal modes are highlighted through orange dotted lines.

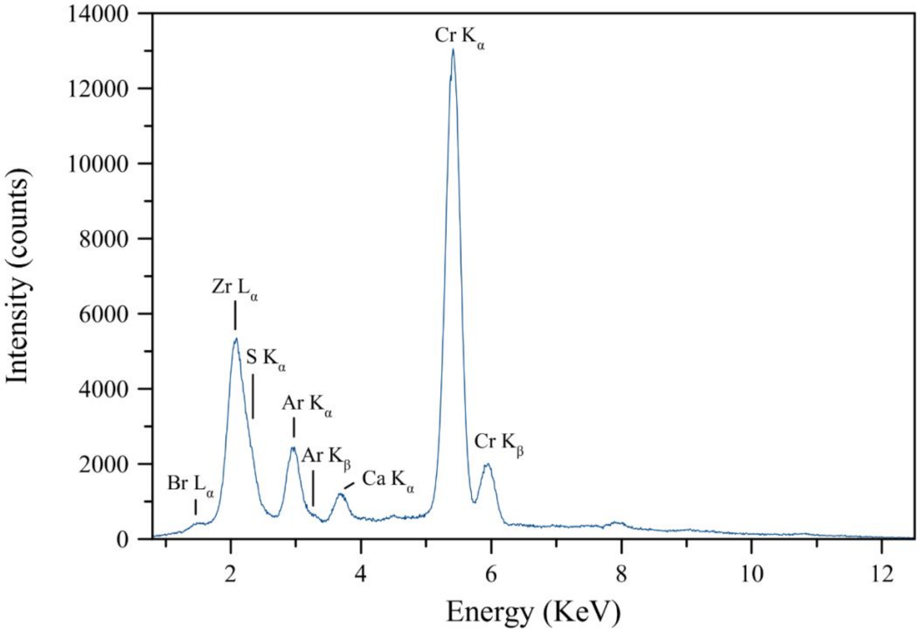

Figure S7. X-ray fluorescence spectrum of **NU-1000-PhTz** measured in air. The characteristic lines of argon come from air, those of chromium from the anode of the X-ray source and those of calcium from the sample-holder.

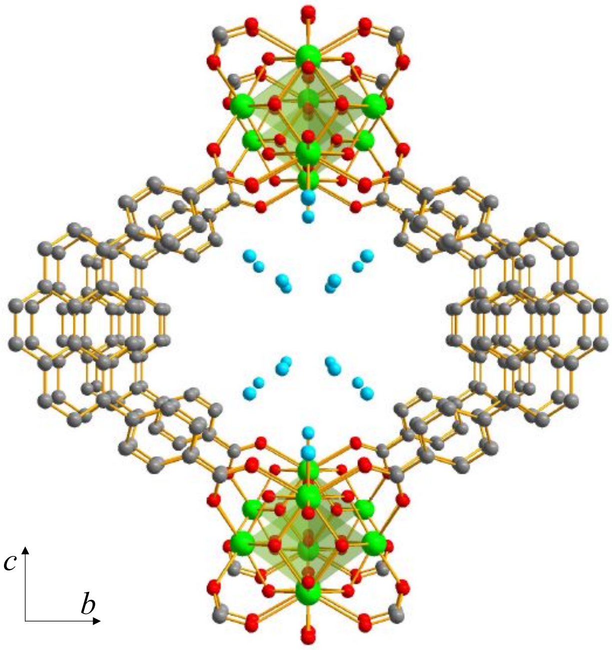

Figure S8. Observed electron density (cyan spheres) in the 8 Å channels of **NU-1000-PhTz**.

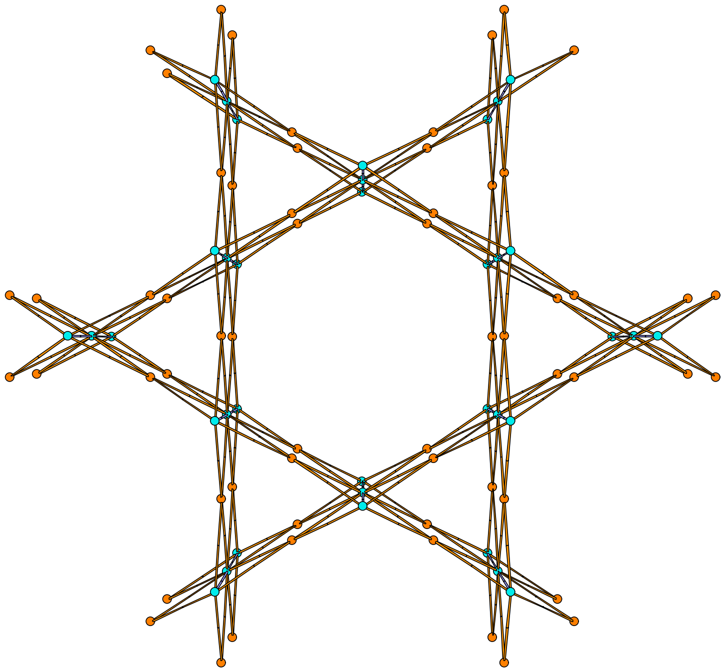

Figure S9. Schematic representation of the **{4,10}-c** topology of **NU-1000-PhTz**. Cyan and orange spheres represent the $[Zr_6]$ clusters and the tetratopic carboxylate ligands, respectively, while the violet sticks represent the **PhTz**$^-$ extra linkers.

Table S2. Main structural parameters of **NU-1000** and **NU-1000-FG** MOFs at comparison.

	a [Å]	*c* [Å]	V [Å^3]	Empty volume [%]	T_{dec} [K]	Ref.
**NU-1000-PhTz**	39.602(2)	16.440(1)	22329(2)	69	820	This work
**NU-1000**[a]	39.3875(7)	16.4829(3)	22145.3	79	800	[7]
NU-1000-NDC-HCl[a]	38.926(3)	16.8351(16)	22091.5	78	773[b]	[8]
F-BA-NU-1000[a]	39.446(2)	16.4166(10)	22122(3)	69	n.a.	[9]

[a] Structure determination at 100 K. [b] Value for the NU-1000-NDC material (NU-1000-NDC-HCl not available).

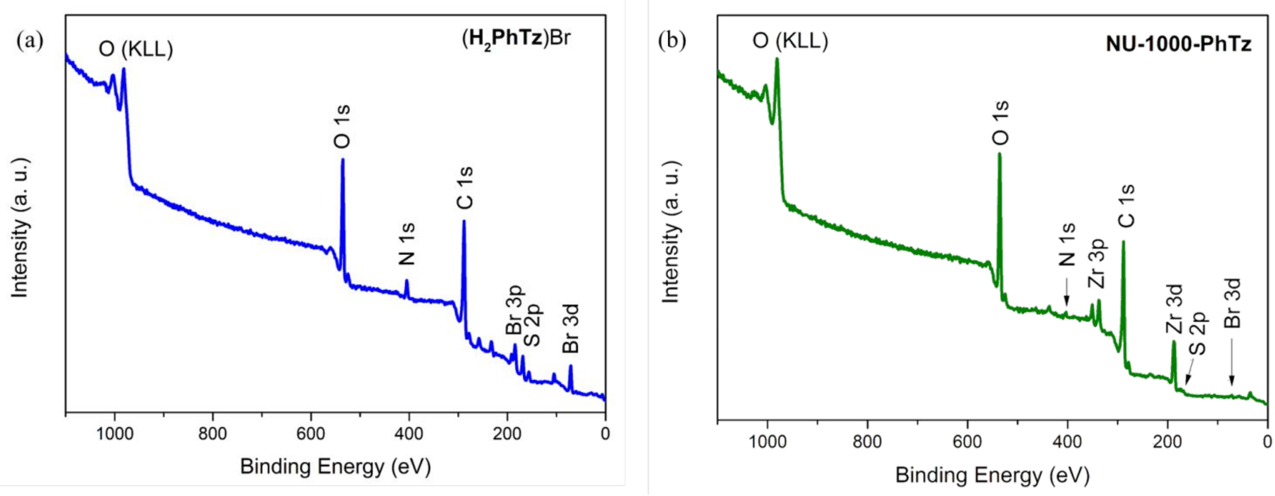

Figure S10. XPS survey scan of (**H$_2$PhTz**)Br (a) and **NU-1000-PhTz** (b).

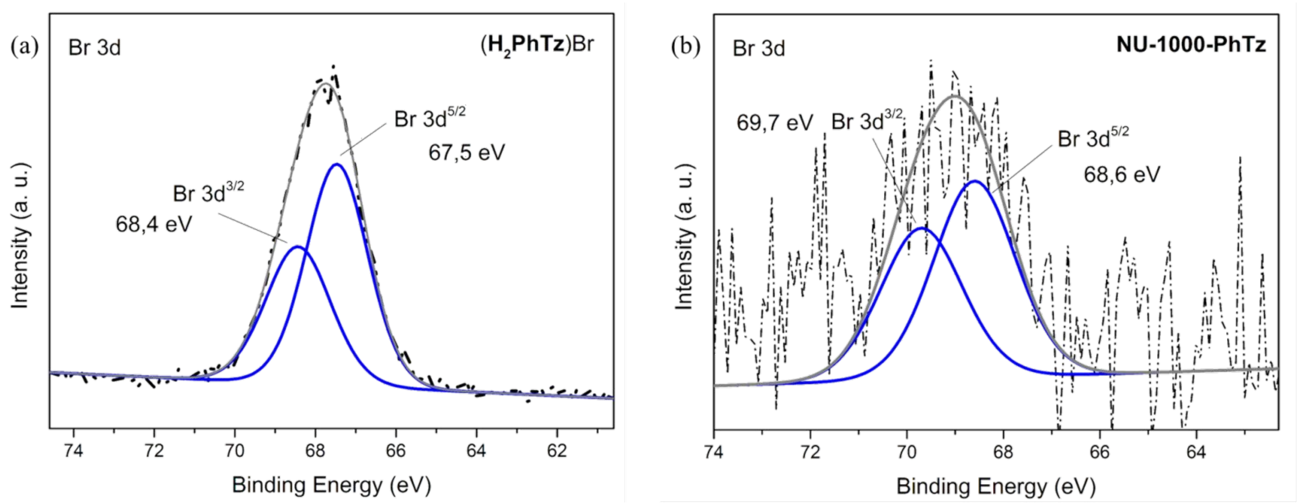

Figure S11. XPS Br *3d* high-resolution spectra of (**H$_2$PhTz**)Br (a) and of **NU-1000-PhTz** (b).

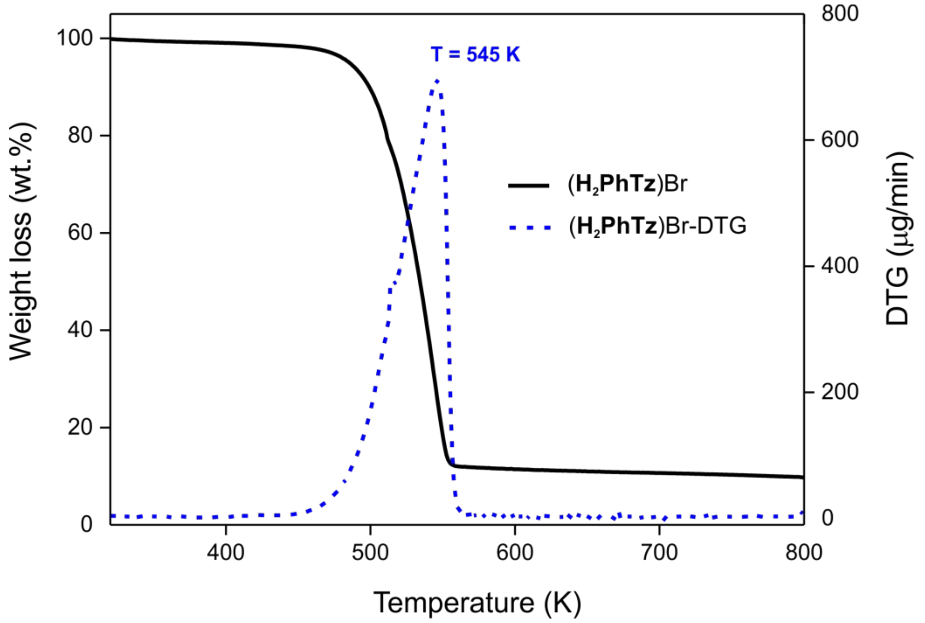

Figure S12. Thermogravimetric analysis *vs.* time profile of (**H_2PhTz**)Br.

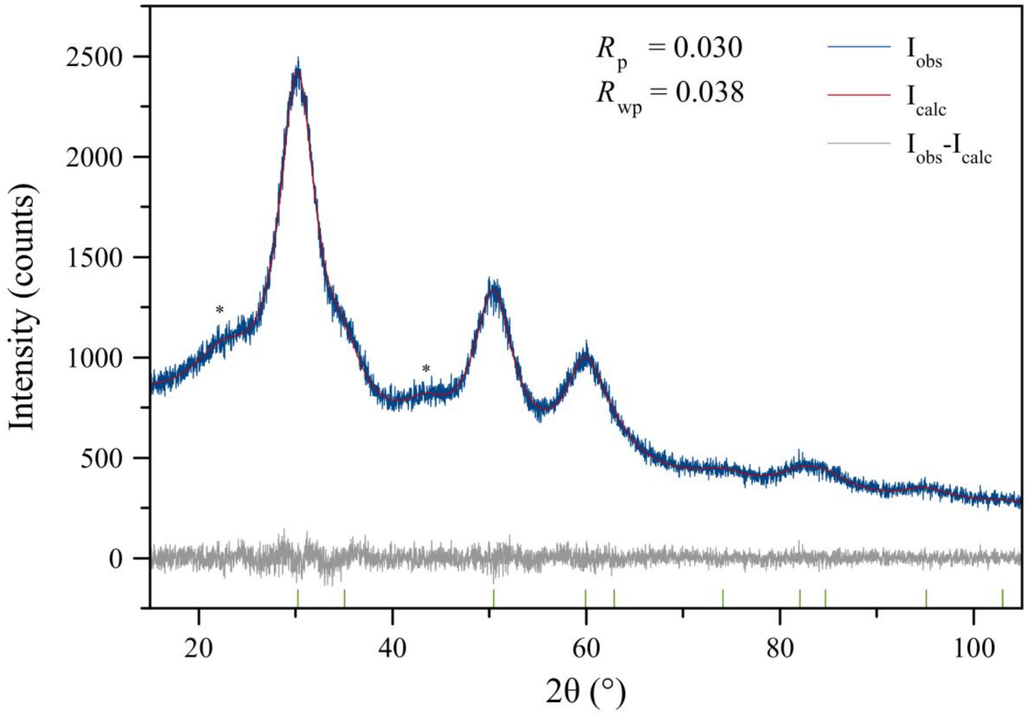

Figure S13. Whole powder pattern refinement carried out with the Le Bail approach on the PXRD pattern of **NU-1000-PhTz** after heating at 1023 K for 15 minutes under N_2 flow, in terms of experimental, calculated and difference traces (blue, red and grey, respectively). The green markers at the bottom indicate the positions of the Bragg reflections. Asterisks are referred to impurities.

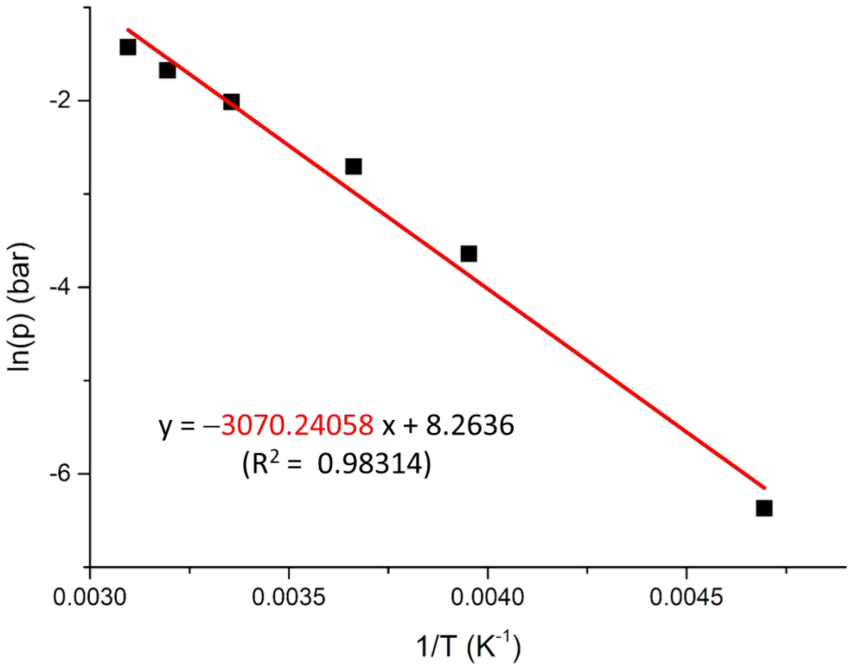

Figure S14. ln(p) *vs.* 1/T Van't Hoff plot for the estimation of the CO_2 isosteric heat of adsorption in **NU-1000-PhTz** through the Clausius-Clapeyron equation.

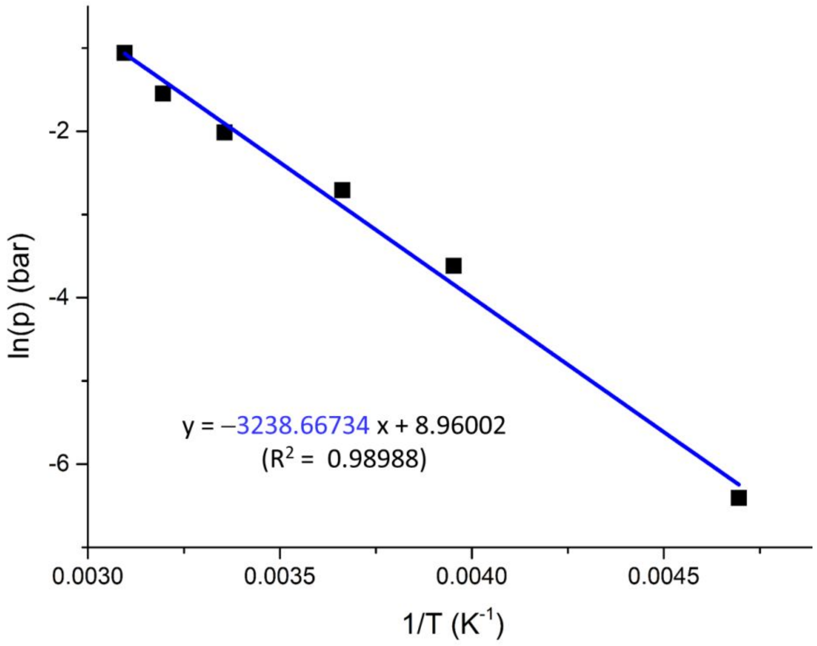

Figure S15. ln(p) *vs.* 1/T Van't Hoff plot for the estimation of the N_2O isosteric heat of adsorption in **NU-1000-PhTz** through the Clausius-Clapeyron equation.

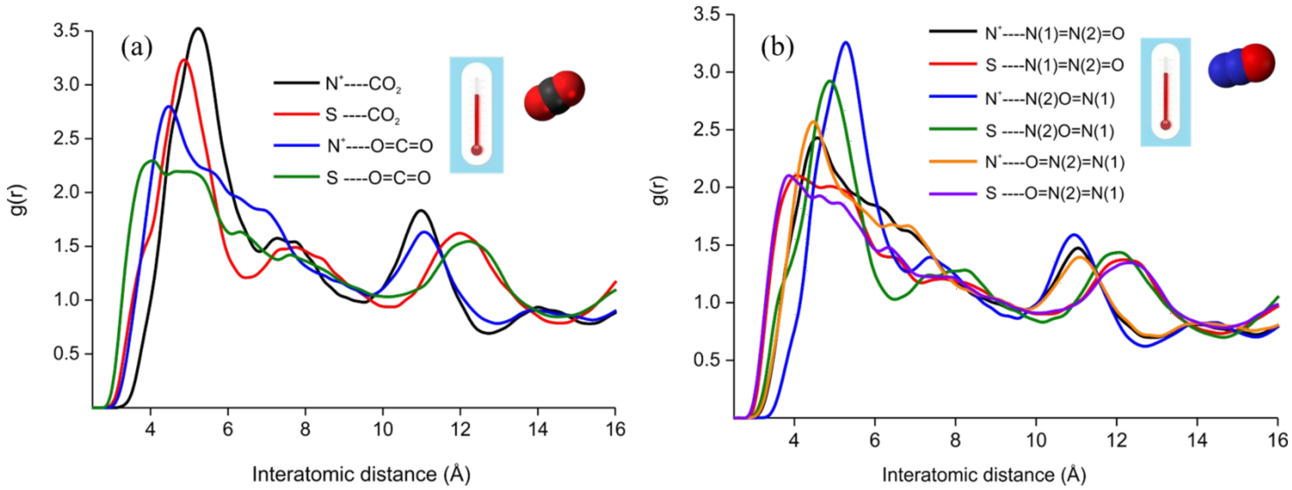

Figure S16. RDF plots at T = 298 K for selected gas-framework interatomic distances for the computed (a) [CO_2@**NU-1000-PhTz**] and (b) [N_2O@**NU-1000-PhTz**] structures.

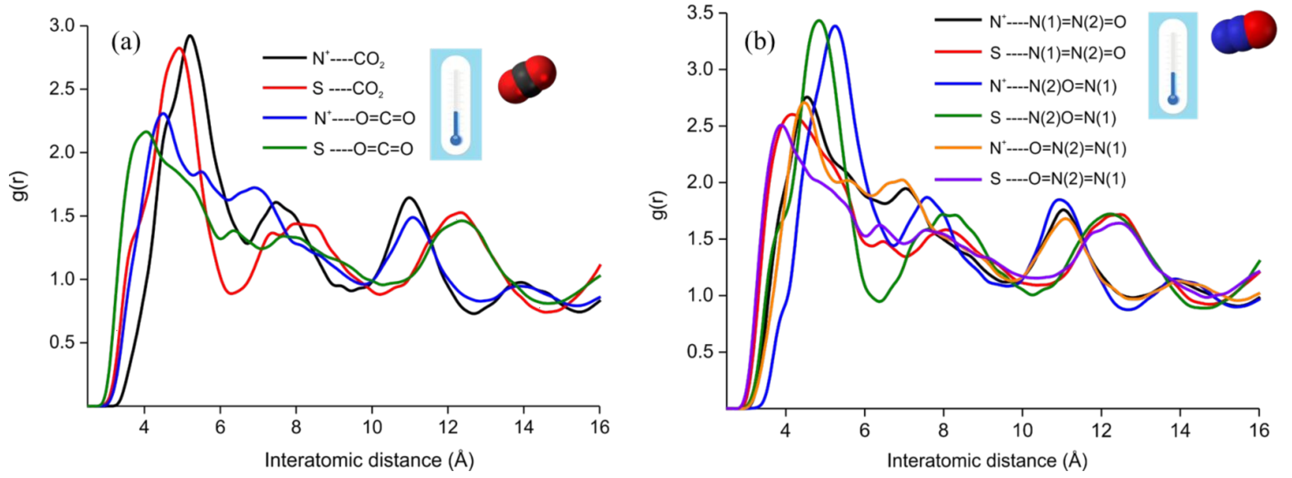

Figure S17. RDF plots at T = 273 K for selected gas-framework interatomic distances for the computed (a) [CO_2@**NU-1000-PhTz**] and (b) [N_2O@**NU-1000-PhTz**] structures.

Table S3. Selected GCMC-optimized interaction distances between CO_2/N_2O and **NU-1000-PhTz**, as derived from the maxima of the RDF plots reported in Figures S14 and S15. The shortest gas-framework distances are highlighted in red.

**Atoms**	**Distance [Å]**	
	**T = 273 K**	**T = 298 K**
S···O=C=O	4.06	**4.06**
N^+···O=C=O	4.49	4.45
S···N^1=N^2=O	4.16	**4.06**
S··· N^2(O)=N^1	4.85	4.88
S···O=N^2=N^1	3.90	**3.86**
N^+···N^1=N^2=O	4.52	4.59
N^+··· N^2(O)=N^1	5.24	5.28
N^+···O=N^2=N^1	4.45	4.49

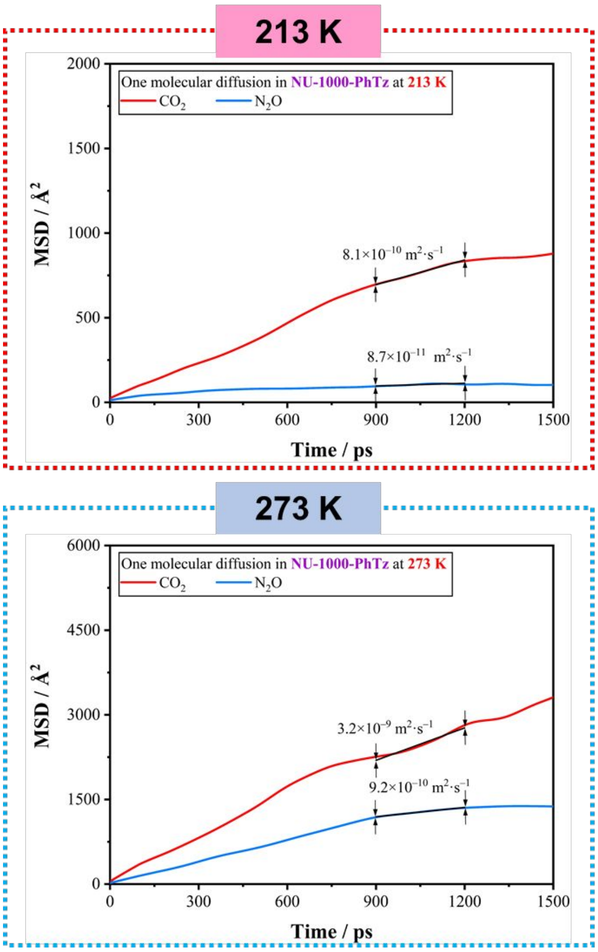

Figure S18. MSD *vs.* time plots of the self-diffusion of N_2O and CO_2 in **NU-1000-PhTz** at variable temperatures. The numbers in the pictures are the diffusion coefficients (Ds).

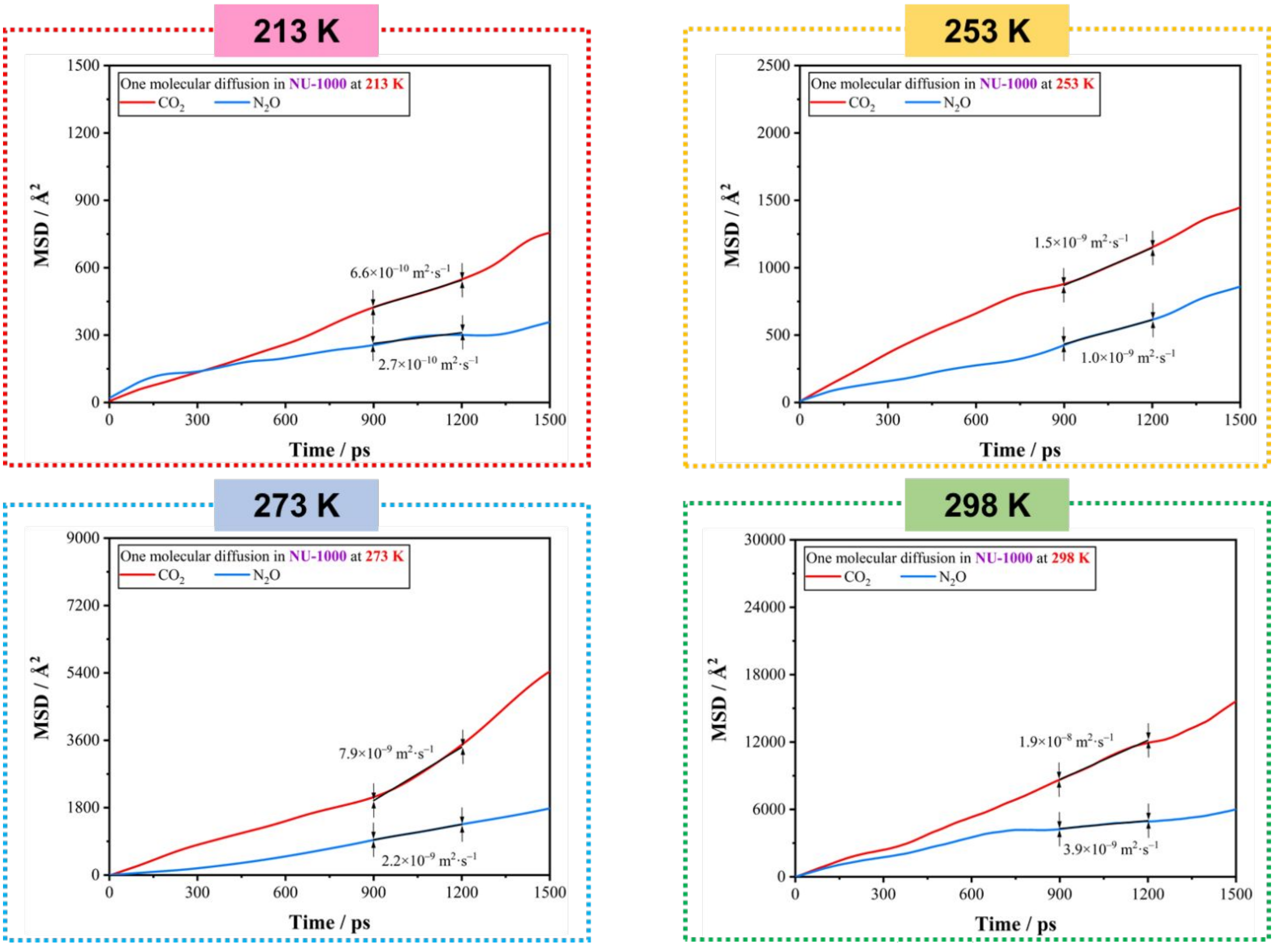

Figure S19. MSD *vs.* time plots of the self-diffusion of N_2O and CO_2 in **NU-1000** at variable temperatures. The numbers in the pictures are the diffusion coefficients (Ds).

Table S4. MD-derived Ds values of CO_2 and N_2O in **NU-1000** and **NU-1000-PhTz** at different temperatures. The ambient temperature values (where a faster N_2O over CO_2 diffusion is observed for the thiazolium-functionalized material) are highlighted in red.

Temperature [K]	Ds [$m^2 \cdot s^{-1}$]			
	NU-1000		NU-1000-PhTz	
	CO_2	N_2O	CO_2	N_2O
213	7.9×10^{-10}	2.7×10^{-10}	8.1×10^{-10}	8.7×10^{-11}
253	1.5×10^{-9}	1.0×10^{-9}	1.5×10^{-9}	2.1×10^{-10}
273	7.9×10^{-9}	2.2×10^{-9}	3.2×10^{-9}	9.2×10^{-10}
**298**	**1.9×10^{-8}**	**3.9×10^{-9}**	**3.1×10^{-9}**	**7.6×10^{-9}**

References

[1] CrysAlis CCD 1.171.31.2 (release 07-07-2006), CrysAlis171.NET, Oxford Diffraction Ltd.

[2] CrysAlis RED 1.171.31.2 (release 07-07-2006), CrysAlis171.NET, Oxford Diffraction Ltd.

[3] Burla, M. C.; Caliandro, R.; Carrozzini, B.; Cascarano, G. L.; Cuocci, C.; Giacovazzo, C.; Mallamo, M.; Mazzone, A.; Polidori, G. Crystal Structure Determination and Refinement *via* SIR2014. *J. Appl. Cryst.* **2015**, *48*, 306-309.

[4] Sheldrick, G. M. Crystal Structure Refinement with SHELXL. *Acta Cryst.* **2015**, *C71*, 3-8.

[5] Nardelli, M. Parst: A System of Fortran Routines for Calculating Molecular Structure Parameters from Results of Crystal Structure Analyses. *Comput. Chem.* **1983**, *7*, 95-98.

[6] Farrugia, L. J. ORTEP-3 for Windows - a Version of ORTEP-III with a Graphical User Interface (GUI). *J. Appl. Crystallogr.* **1997**, *30*, 565.

[7] Mondloch, J. E.; Bury, W.; Fairen-Jimenez, D.; Kwon, S.; DeMarco, E. J.; Weston, M. H.; Sarjeant, A. A.; Nguyen, S. T.; Stair, P. C.; Snurr, R. Q.; Farha, O. K.; Hupp, J. T. Vapor-Phase Metalation by Atomic Layer Deposition in a Metal–Organic Framework. *J. Am. Chem. Soc.* **2013**, *135*, 10294-10297.

[8] Peters, A. W.; Otake, K.; Platero-Prats, A. E.; Li, Z.; DeStefano, M. R.; Chapman, K. W.; Farha, O. K.; Hupp, J. T. Site-Directed Synthesis of Cobalt Oxide Clusters in a Metal–Organic Framework. *ACS Appl. Mater. Interfaces* **2018**, *10*, 15073-15078.

[9] Liu, J.; Li, Z.; Zhang, X.; Otake, K.; Zhang, L.; Peters, A. W.; Young, M. J.; Bedford, N. M.; Letourneau, S. P.; Mandia, D. J.; Elam, J. W.; Farha, O. K.; Hupp, J. T. Introducing Nonstructural Ligands to Zirconia-like Metal–Organic Framework Nodes To Tune the Activity of Node-Supported Nickel Catalysts for Ethylene Hydrogenation. *ACS Catal.* **2019**, *9*, 3198-3207.
